# Dental biofilms contain DNase I-resistant Z-DNA and G-quadruplexes but alternative DNase overcomes this resistance

**DOI:** 10.1038/s41522-025-00694-x

**Published:** 2025-05-19

**Authors:** Dominique C. S. Evans, Mathilde F. Kristensen, Gabriel Antonio S. Minero, Lorena G. Palmén, Inge Knap, Manish K. Tiwari, Sebastian Schlafer, Rikke L. Meyer

**Affiliations:** 1 Interdisciplinary Nanoscience Centre, Aarhus University, Aarhus, Denmark; 2 Novonesis A/S, Bagsværd, Denmark; 3 Department of Dentistry and Oral Health, Section for Oral Ecology, Cariology, Aarhus University, Aarhus, Denmark; 4 Department of Biology, Aarhus University, Aarhus, Denmark

**Keywords:** Bacteria, Biofilms, Plaque

## Abstract

Extracellular DNA (eDNA) in bacterial biofilms can form non-canonical structures like Z-DNA and G-quadruplex (G4), which enhance biofilm resilience by providing protection against mammalian DNases. However, the conformation of eDNA in dental biofilms remains unexplored. Using fluorescence immunolabeling and confocal microscopy, we examined dental biofilms from healthy and caries-active subjects, revealing B-DNA, G4-, and Z-DNA structures surrounding clusters of bacteria, with some structures directly associated with the bacterial cell surface. We demonstrated that these non-canonical DNA structures were resistant to mammalian DNase I. Using a *Streptococcus mutans* biofilm model, we visualised fluorescently labelled eDNA during enzyme treatment and identified both an experimental nuclease and a DNase I-chloroquine combination capable of removing eDNA that was resistant to DNase I. These findings suggest that G4 and Z-DNA structures represent novel targets for improved enzyme formulations in controlling dental biofilms and potentially other biofilms containing these secondary DNA structures.

## Introduction

Dental biofilms are complex and diverse microbial communities encased in a protective extracellular matrix comprised of polysaccharides, nucleic acids, proteins, lipids, and inorganic ions^1^. When left uncontrolled, dental plaque can cause gingivitis, periodontitis, and caries^2,3^. Caries is a major global health problem estimated to affect 3 billion people worldwide^4^ and disproportionately affects people with lower socioeconomic status^5^. In addition to these oral diseases, poor oral health is also associated with several other systemic diseases such as coronary artery disease and rheumatoid arthritis^2,6^.

Enzymes that target and break down the extracellular matrix show potential for controlling dental biofilms without disturbing the healthy microbiota and hence for promoting oral health^7^. Glucanohydrolases, such as dextranases and mutanases, target the polysaccharide matrix and impact dental biofilms^8–14^. Other enzymes that have been tested include lysozyme, beta-glucanases, lipases, proteases, and DNase I^9,10,12,14–19^. Many of these studies report successful prevention and removal of dental biofilms, especially with combinations of enzymes that target different matrix components simultaneously.

Many studies focus on the removal of polysaccharides for dental biofilm control, but eDNA is also a major dental biofilm component^19–23^ and is thus a potential target for enzyme-based biofilm control. It is well documented that DNase I removes young biofilms but is ineffective against mature biofilms^24^. This has been shown for many types of biofilm^25^, including dental biofilms^15,19,26,27^. One reason that can explain this discrepancy was only recently uncovered and involves the presence of DNA structures that are resistant to DNase I, an endonuclease with a preference for cleaving at AT base pairs in dsDNA, and with some (although strongly reduced) activity for cleaving ssDNA and DNA-RNA hybrids.

Extracellular DNA in biofilms originates from genomic DNA from the bacteria or immune cells. It was therefore thought to exist as B-DNA: the canonical right-handed double helix characterised by Watson-Crick base pairing, which is sensitive to DNase I^28^. However, DNA can also adopt non-canonical secondary structures, such as Z-DNA, G-quadruplexes (G4), i-Motifs, and triplex-DNA. Among these structures, Z-DNA and G4 can be abundant in biofilms^29–31^.

Z-DNA is a left-handed double helix that preserves Watson-Crick base pairing and resists degradation by DNase I^32^. It is stabilised in biofilms by DNA-binding proteins belonging to the DNABII family, and Z-DNA has been detected in a variety of biofilms in vivo and in vitro^29,30,33^. G4 structures are characterised by non-canonical Hoogesteen base pairing in nucleotide regions rich in guanine. G4-DNA and -RNA have been detected in vitro in biofilms formed by *Pseudomonas aeruginosa*^31^, *Staphylococcus epidermidis*^29^, and in vivo in an implant-associated *Staphylococcus aureus* infection^29^. G4 structures also resist degradation by DNase I^29^.

The discovery of non-canonical DNA structures in biofilms is so recent that only a few studies have investigated if oral microorganisms produce such structures in the biofilm matrix. Z-DNA has been found in the matrix of *Streptococcus mutans* biofilms and in dental calculus^30,33^, but it is currently unknown whether G4 and Z-DNA structures exist in in vivo-grown dental biofilms. We hypothesised that these structures are present in dental biofilms and that they impede biofilm removal by DNase I. In this study, we investigate the presence of G4 and Z-DNA in dental biofilms from healthy and caries-active subjects, and we thereby assess whether these are relevant targets for improved dental biofilm control.

## Results

### G4 and Z-DNA are present in dental biofilms from healthy subjects

**Fig. 1:**
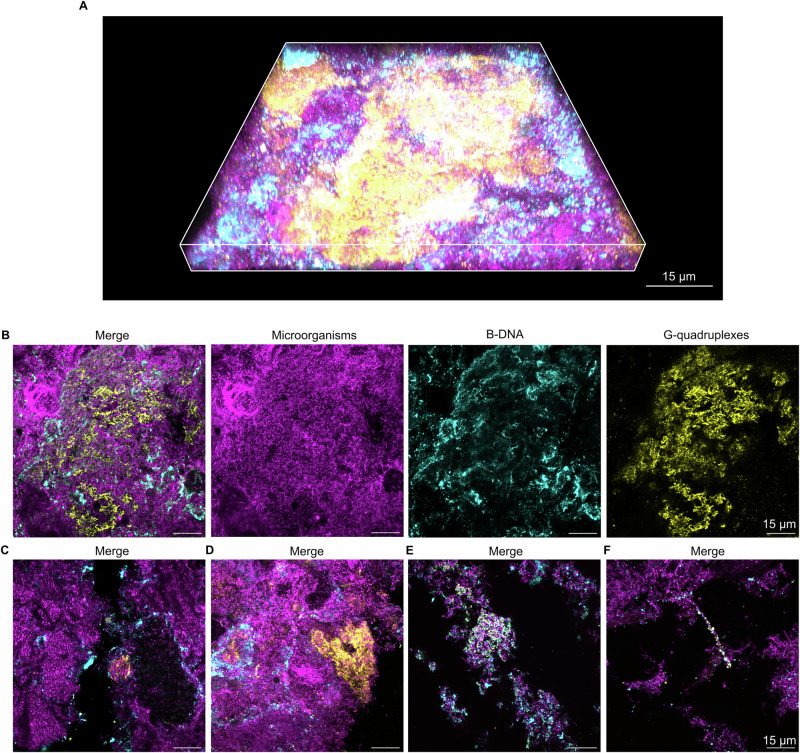
Extracellular G4 structures in dental biofilms from healthy subjects. Confocal microscopy images show microorganisms (FM4-64 membrane stain, magenta), B-DNA (anti-dsDNA antibody, cyan), and G4 (BG4 antibody, yellow) in dental biofilms. **A** 3D merged confocal image of an example of a G4 structure in a dental biofilm from a healthy subject. **B** Single channel and merged 2D confocal images of G4 structures in a dental biofilm from a healthy subject. **C**–**F** Merged 2D images showing further examples of G4 in dental biofilms from healthy subjects. The image in (**A**) was prepared in Zen Blue (Zeiss) and the images in (**C**–**F**) are images of single planes in biofilms that were prepared in Fiji ImageJ^53^.

**Fig. 2:**
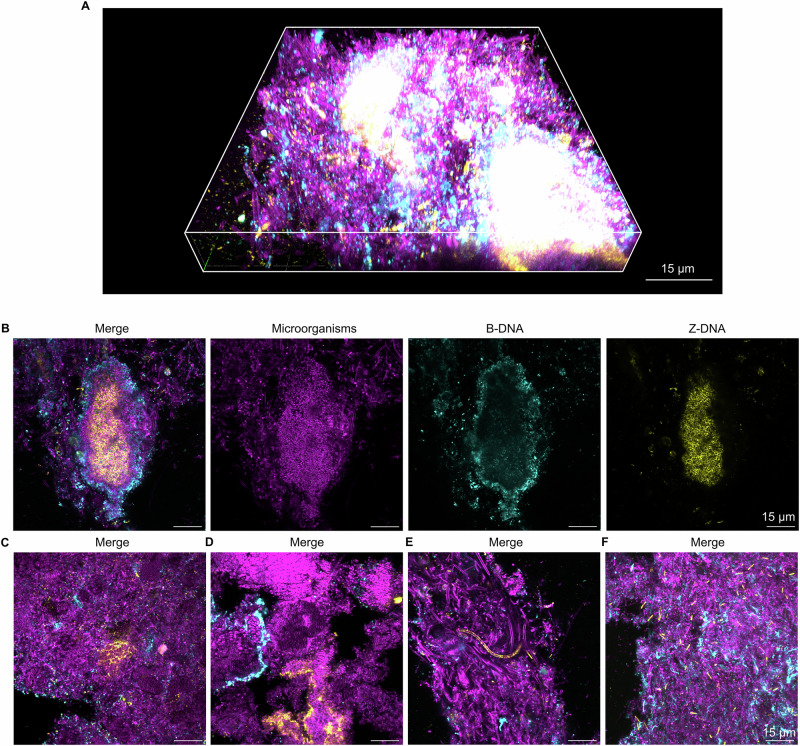
Extracellular Z-DNA structures in dental biofilms from healthy subjects. Confocal microscopy images show microorganisms (FM4-64 membrane stain, magenta), B-DNA (anti-dsDNA antibody, cyan), and Z-DNA (Z22 antibody, yellow) in dental biofilms. **A** 3D merged confocal image of an example of a Z-DNA structure in a dental biofilm from a healthy subject. **B** Single channel and merged 2D confocal images of a Z-DNA structure in a dental biofilm from a healthy subject. **C**–**F** Merged 2D images showing further examples of G4 in dental biofilms from healthy subjects. The image in (**A**) was prepared in Zen Blue (Zeiss) and the images in (**C**–F) were prepared in Fiji ImageJ^53^. **C**–**E** are images of single planes within biofilms, and (**F**) is a maximum intensity Z-projection.

We used fluorescence immunolabelling to visualise G4, Z-DNA, and B-DNA in dental biofilms from healthy subjects to assess whether non-canonical DNA structures are present in habitual plaque (Figs. 1, 2). We detected large areas of eDNA that were rich in G4 structures surrounding clusters of microorganisms (Fig. 1A–E) in dental biofilms from 6 out of 10 subjects, and large eDNA structures containing Z-DNA in 9 out of 10 subjects (Fig. 2A–D). There was considerable variation in the abundance of eDNA and non-canonical DNA structures within a given plaque sample and between samples from different individuals. For most subjects, the structures represented a small fraction of the total biofilm, although a few samples appeared to contain large amounts. Quantification was not possible due to the large sample heterogeneity, and we therefore chose to qualitatively assess the appearance of the biofilms.

eDNA containing G4 or Z-DNA appeared to be associated to clusters of microorganisms (Figs. 1B, C, 2B, C). Some clusters were surrounded by concentric rings of eDNA formed by an inner layer of Z-DNA or G4 and an outer layer of B-DNA (Figs. 1B, D, 2A, B). Other clusters were surrounded by a ring of G4, Z-DNA, or B-DNA alone and not in combination with other eDNA structures (Figs. 1D, 2C, D). We are critical as to whether the antibodies used to visualise the different structures were able to fully penetrate the biofilm; thus, fluorescence around the rim of bacterial clusters could indicate that antibodies only bound the outer layers of the biofilm matrix. However, since B-DNA was always the outermost layer, it would imply that the antibody used to visualise B-DNA penetrated biofilms less than other antibodies, which is unlikely.

In addition to eDNA in clusters of bacteria, we also observed individual bacteria with G4 or Z-DNA on the cell surface. These were present in plaque from 7 out of 10 subjects for G4, and in 9 out of 10 subjects for Z-DNA. In general, individual microorganisms with surface-associated secondary DNA structures were either small rods or were very large and long cells that resembled fungal hyphae (Figs. 1F, 2E, F).

### G4 and Z-DNA are present in dental biofilms from caries-active subjects

**Fig. 3:**
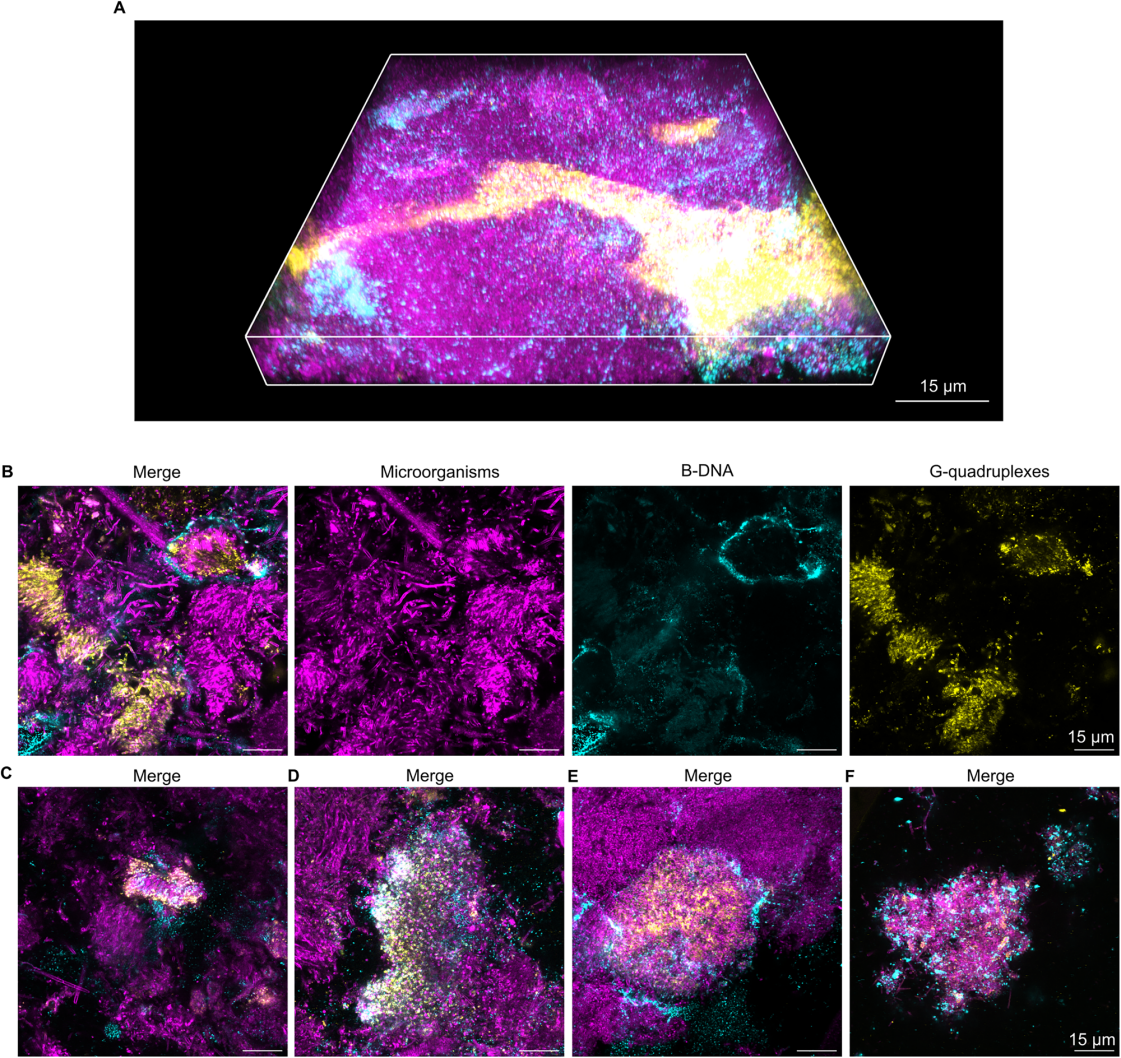
Extracellular G4 structures in dental biofilms from caries-active patients. Confocal microscopy images show microorganisms (FM4-64 membrane stain, magenta), B-DNA (anti-dsDNA antibody, cyan), and G4 (BG4 antibody, yellow) in dental biofilms from caries-active subjects. **A** Merged 3D image of an example G4 structure in a dental biofilm from a caries-active subject. **B** Single channel and merged 2D images of G4 structures in a dental biofilm from a caries-active subject. **C**–**F** Merged 2D images of G4 structures in dental biofilms from caries-active subjects. The image in (**A**) was prepared in Zen Blue (Zeiss) and the images in (**C**–**F**) are images of single planes in biofilms that were prepared in Fiji ImageJ^53^.

**Fig. 4:**
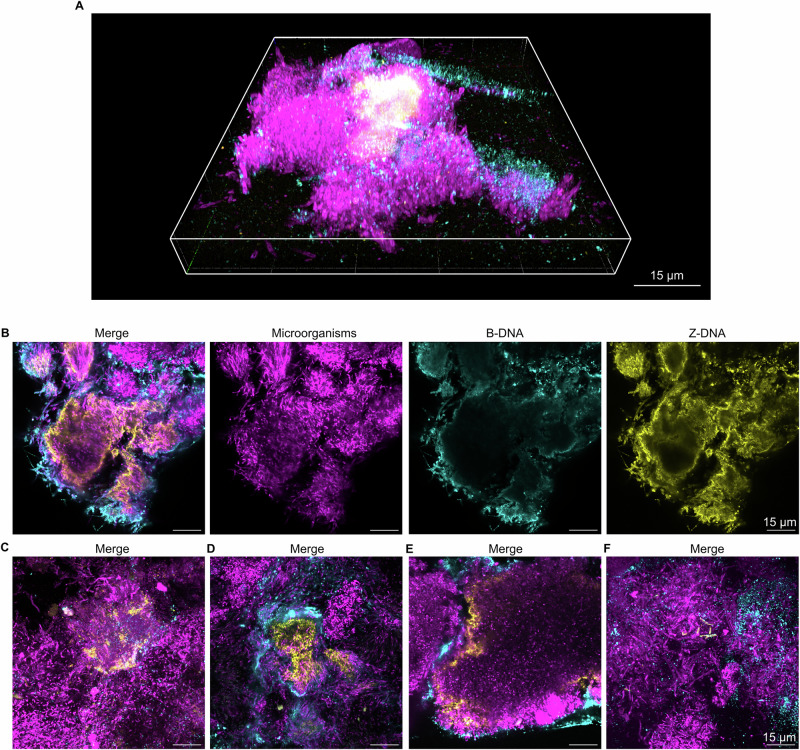
Extracellular Z-DNA structures in dental biofilms from caries-active patients. Confocal microscopy images show microorganisms (FM4-64 membrane stain, magenta), B-DNA (anti-dsDNA antibody, cyan), and Z-DNA (Z22 antibody, yellow) in dental biofilms from caries-active subjects. **A** Merged 3D image of an example Z-DNA structure in a dental biofilm from a caries-active subject. **B** Single channel and merged 2D images of a Z-DNA structure in a dental biofilm from a caries-active subject. **C**–**F** Merged 2D images of Z-DNA structures in dental biofilms from caries-active subjects. The image in (**A**) was prepared in Zen Blue (Zeiss) and the images in (**C**–**F**) are images of single planes in biofilms that were prepared in Fiji ImageJ^53^.

Dental biofilms from healthy and caries-active subjects have different microbial compositions, and we therefore investigated if G4 and Z-DNA are also associated with dental biofilms from caries-active subjects. We collected dental biofilms from 10 patients with caries, and visualised G4, Z-DNA, and B-DNA via immunolabelling (Figs. 3, 4).

Again, we detected large clusters of microorganisms with eDNA containing G4 (Fig. 3A–E) and Z-DNA (Fig. 4A–E). Biofilms from 6 out of 10 caries-active subjects contained patches of eDNA with G4, and biofilms from 7 out of 10 individuals contained Z-DNA. Similar to dental biofilms from healthy subjects, some bacterial clusters contained eDNA in concentric rings with an outer layer of B-DNA and an inner layer of G4 or Z-DNA (Figs. 3A, E, 4B, D, E), while other clusters showed a more homogenous distribution of eDNA (Figs. 3B, 4C). In other areas of the biofilm, G4 and Z-DNA were associated with the surface of bacteria. Samples where heterogenous and the patches of eDNA only represent a small fraction of the biofilm.

### G4 and Z-DNA structures remain in dental biofilms after treatment with DNase I

DNase I is often used to degrade eDNA in biofilms, but it is ineffective at removing mature biofilms^24^, possibly due to the resistance of non-canonical DNA structures to degradation by DNase I^29,30^. We investigated whether DNase I could degrade non-canonical DNA structures in dental biofilms by comparing G4, Z-DNA, and B-DNA in dental biofilms from the same subject after treatment with DNase I or buffer. The subject belonged to the group of 10 participants without clinical signs of caries or periodontitis. We could not compare the same fields of view (FOV) before and after treatment because the antibodies used for immunolabelling would protect the DNA from degradation by DNase I. We therefore compared the presence of G4 and Z-DNA in arbitrarily chosen FOV in separate samples (taken from the same subject) with and without DNase I treatment.

**Fig. 5:**
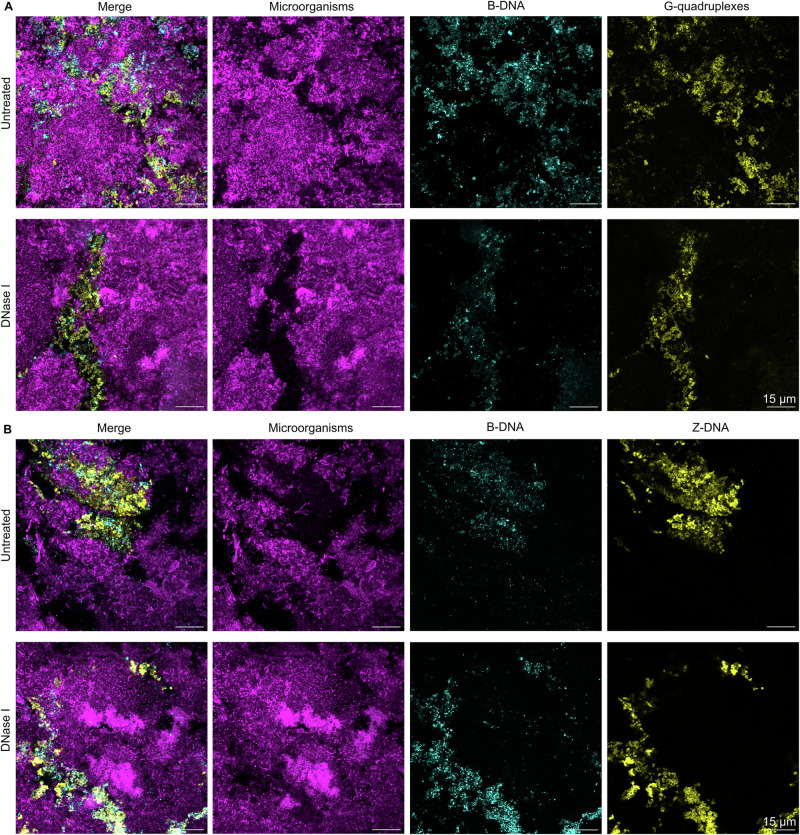
Extracellular DNA structures remain in dental biofilms after treatment with DNase I. Confocal microscopy images show microorganisms (FM4-64 membrane stain, magenta), B-DNA (anti-dsDNA antibody, cyan), and Z-DNA (Z22 antibody, yellow) or G4 (BG4 antibody, yellow) in dental biofilms from a healthy subject visualised by confocal microscopy. **A** Single channel and merged 2D images of G4 in dental biofilms with and without treatment with DNase I. **B** Single channel and merged 2D images of Z-DNA in dental biofilms with and without treatment with DNase I. G4, Z-DNA, and also B-DNA structures remained in the dental biofilms after a 1 h treatment with DNase I. All images in this figure were collected using the same microscope settings, and the brightnesses of all images were adjusted equally in Fiji ImageJ^53^.

Both G4 and Z-DNA structures were present in the dental biofilm regardless of whether the sample was treated with DNase I (Fig. 5). This indicates that G4 and Z-DNA are unaffected by treatment with DNase I. It was expected that DNase I would remove B-DNA in the biofilms, but interestingly, the signal from B-DNA appeared very similar in the samples with and without DNase I treatment and was located in regions where it overlapped with either G4 or Z-DNA structures. Perhaps B-DNA was also protected from degradation by these secondary structures or by another matrix component. This result is consistent with observations by Buzzo et al.^30^ who treated biofilms with DNase I and did not observe that B-DNA was completely removed, but rather that the ratio between Z-DNA and B-DNA increased. We confirmed that DNase I was active against DNA in a separate experiment (Supplementary Fig. 1), therefore, this indicates that DNase I fails to remove B-DNA from these biofilms.

### *Streptococcus mutans* aggregate biofilm model is rich in eDNA, G4, and Z-DNA

After analysis of dental plaque indicated that DNase I could not remove G4 and Z-DNA, we wanted to develop an orally relevant biofilm model which was suitable to quantify the removal of these recalcitrant DNA structures by different types of nucleases. We assessed biofilms inoculated from dental plaque or as single-species biofilms of *Lactobacillus paracasei, Lactobacillus rhamnosus, Fusobacterium nucleatum, Porphyromonas gingivalis, Prevotella intermedia, Streptococcus mitis, Streptococcus oralis, Streptococcus gordonii,* and *Streptococcus mutans*. Biofilms were grown in microwell plates under many different growth conditions (Supplementary Table 1) and examined for the presence of eDNA using SYTOX Green staining, and the presence of Z-DNA and G4 by fluorescence immunolabeling. While we did find some non-canonical DNA structures in biofilms inoculated with plaque (Supplementary Fig. 2), *P. intermedia* (Supplementary Fig. 2), *P. gingivalis* (Supplementary Fig. 3), and *S. mutans* (Supplementary Fig. 4), the abundance of eDNA and the occurrence of non-canonical structures was highly variable among replicates. Only when we sampled biofilms directly from agar, or inoculated biofilms directly from agar, dense eDNA-rich *S. mutans* biofilms packed with G4 appeared (Supplementary Fig. 4H, I). We therefore concluded that the solid/liquid interface biofilms fostered a different biofilm phenotype that was unlike the phenotype observed in plaque.

**Fig. 6:**
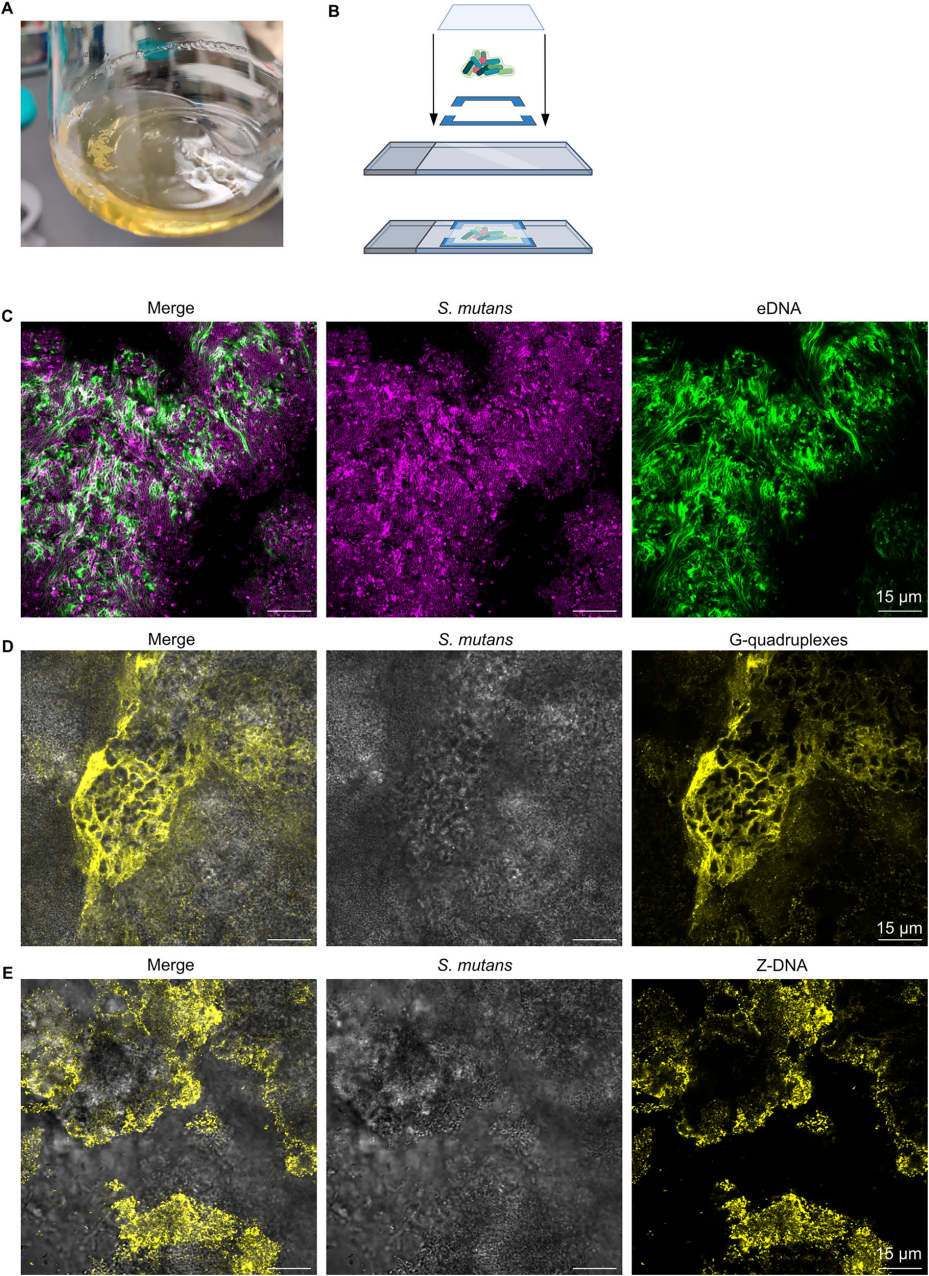
eDNA rich *S. mutans* aggregate biofilm model. **A** *S. mutans* aggregate biofilms grown in brain heart infusion medium supplemented with 1 % sucrose and 200 mM NaCl. **B** Diagram representing how *S. mutans* aggregate biofilms were immobilised in a sandwich chamber. Panel B was created in BioRender. Evans, D. (2025) https://BioRender.com/j96w607. **C** Representative 2D confocal image of an *S. mutans* aggregate biofilm with eDNA. Cells are stained with FM 4-64 (magenta), and eDNA is stained with SYTOX Green (green). **D** *S. mutans* aggregate biofilm (brightfield, grey) with G4 (BG4 antibody, yellow). **E** *S. mutans* aggregate biofilm (brightfield, grey) with Z-DNA (Z22 antibody, yellow). The brightnesses of each image were adjusted individually in Fiji ImageJ^53^.

We noticed that *S. mutans* forms suspended aggregates, i.e. suspended biofilms (Fig. 6A). We sampled these suspended aggregates (henceforth called “aggregate biofilms”) for microscopy (Fig. 6B), and discovered that they were rich in eDNA (Fig. 6C). These biofilms also contained G4 structures (Fig. 6D) and Z-DNA (Fig. 6E), and the phenotype was consistent across replicate experiments. We noted that Z-DNA was mostly located on the surface of the aggregates.

### *S. mutans* aggregate biofilm model contains DNase I-resistant eDNA

**Fig. 7:**
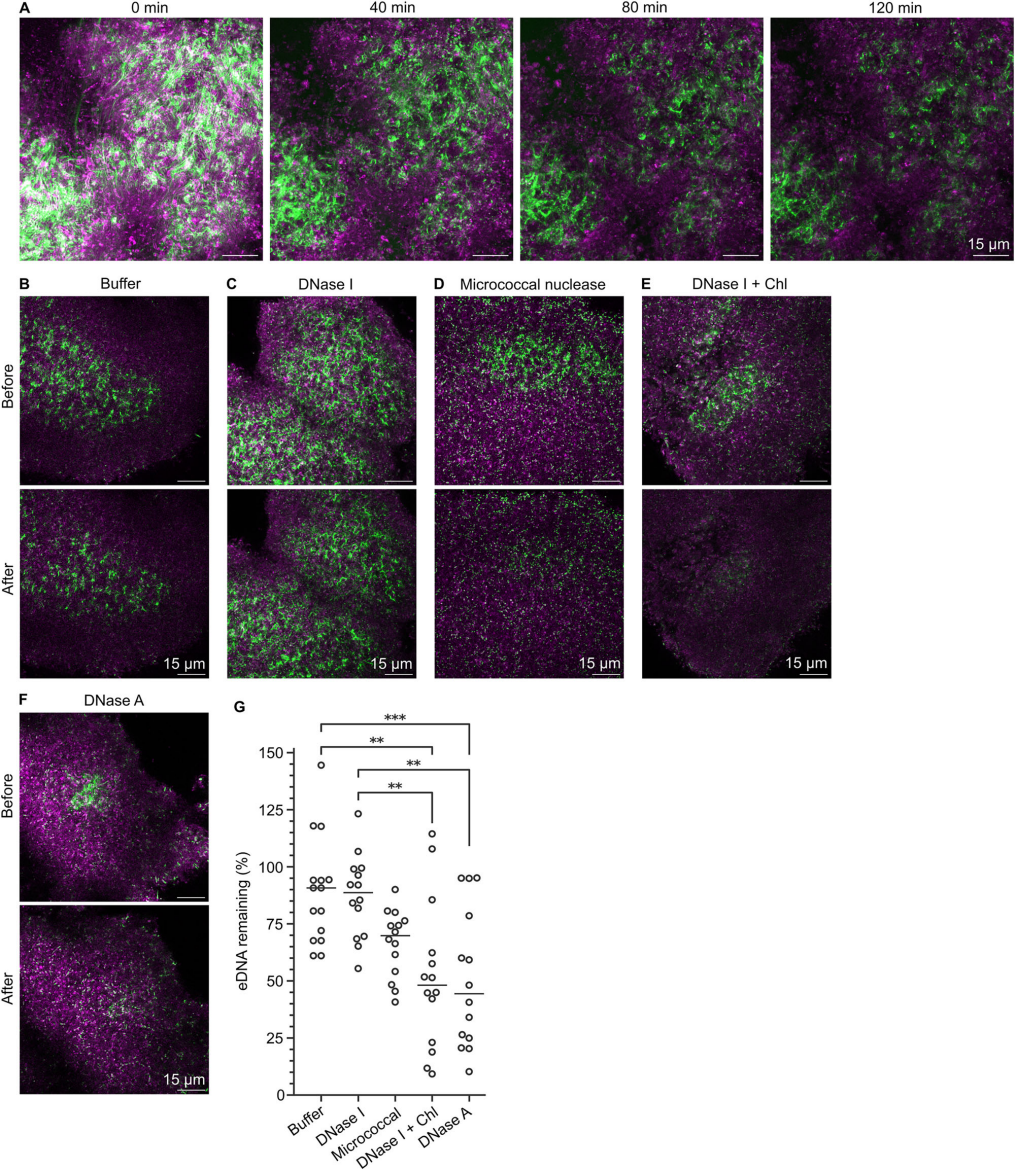
Enzyme removal of recalcitrant eDNA in *S. mutans* biofilms. **A** *S. mutans* aggregate biofilm (FM 4-64, magenta) treated with DNase I for 120 min and imaged every 2 min with time-lapse confocal microscopy. Pockets of eDNA (SYTOX Green, green) that were recalcitrant to DNase I remained after 120 min incubation. A maximum intensity Z-projection of a 5.3 μm Z-stack is shown. In subsequent experiments, *S. mutans* biofilms were pre-treated for 120 min with DNase I, followed by a second 60 min treatment with (**B**) buffer, **C** DNase I, **D** micrococcal nuclease, **E** DNase I combined with chloroquine (Chl), and an experimental DNase (**F**) DNase A. Biofilms were visualised before and after the subsequent treatment. Analysis was performed on 5 2D FOV from each sample. Experiments were performed in triplicate under identical conditions and imaging settings. **G** Fraction of DNase I-recalcitrant eDNA remaining after each enzyme treatment. The technical variation was greater than the biological variation therefore all replicates were treated as true replicates. Each point corresponds to results from 1 FOV and black bars represent group medians. The data were tested for normality, then analysed with a one-way ANOVA followed by a Tukey’s test. *** denotes *p* < 0.001 and ** denotes *p* < 0.01 significance levels. The brightnesses of each image presented were adjusted in Fiji ImageJ^53^. The adjustments were identical for the “before” and “after” images (**B**–**F**) and for all timepoints (**A**).

The consistent phenotype made the *S. mutans* aggregate biofilms suitable for assessment of DNase activity against non-canonical DNA structures in biofilms. To address this, we performed time-lapse confocal microscopy to visualise eDNA in real-time during DNase treatment. We verified that fluorescent labelling of DNA with SYTOX Green did not interfere with DNase activity in a preliminary experiment (Supplementary Fig. 5), and then used SYTOX Green for real-time eDNA imaging. Much of the eDNA was removed after about 1 h incubation with DNase I, but pockets of recalcitrant eDNA remained (Fig. 7A, Supplementary Movie 1). The eDNA that was removed within the first 1 h was most likely DNase I-sensitive B-DNA, and the recalcitrant eDNA could be protected from degradation by DNase I possibly by adopting a secondary conformation such as G4 or Z-DNA. We therefore turned our attention to determine if other nucleases could remove this DNase I-resistant eDNA.

### A new experimental DNase removes recalcitrant DNA in *S. mutans* biofilms

We hypothesised that the recalcitrant eDNA in *S. mutans* aggregate biofilms resisted DNase I due to the presence of G4 and Z-DNA, which was previously shown to resist degradation by this enzyme^29,30^. We therefore investigated if other nucleases could degrade these non-canonical DNA structures. Using an in vitro assay with oligonucleotides in the B, G4 and Z conformation^29^, we identified an experimental DNase (DNase A) with activity against G4 and Z-DNA oligos (Table 1). We then tested the activity of DNase A, DNase I, and micrococcal nuclease against the recalcitrant eDNA in *S. mutans* aggregate biofilms. Micrococcal nuclease is an endo-exonuclease from *Staphylococcus aureus* that also has activity against G4^29^. Finally, we hypothesised that combining DNase I with an adjuvant (chloroquine) that converts Z-DNA into B-DNA^30^ would improve the removal of eDNA from *S. mutans* aggregate biofilms.

**Table 1:** Comparison of commercial micrococcal nuclease, DNase I, and an experimental nuclease (DNase A) based on their performance cleaving two synthetic G4 and Z-DNA substrates

Enzyme	Remaining G4 (%)		Remaining Z-DNA (%)	
	Oligo Tel	Oligo C-myc	Oligo B1-B1c	Oligo B2-B2c
Micrococcal nuclease	−1.60 ± 1.02	3.61 ± 4.61	103 ± 2.52	135 ± 8.44
DNase I	97.4 ± 3.93	94.2 ± 3.45	78.0 ± 2.83	106 ± 10.9
DNase A	25.0 ± 28.0	25.8 ± 18.4	0.08 ± 0.03	0.22 ± 0.15

The quantification of enzymatic cleaving of substrates is based on fluorescence of DNA-binding dyes SYTO60 and Picogreen, respectively, added at the end of enzymatic treatment.

In these experiments, we first pre-treated biofilms with DNase I for 2 h to remove B-DNA, and then visualised FOV containing recalcitrant eDNA before and after a second 1 h enzyme treatment. We quantified the effect of the enzyme treatment as changes in the area coverage of eDNA. To visualise the same FOV before and after the second enzyme treatment, we immobilised *S. mutans* biofilms in a sandwich chamber (Fig. 6B) that had an inlet and outlet that allowed us to exchange the medium that surrounded the biofilms without losing the FOV. In each experiment, we visualised 5 FOV, and we performed each experiment in triplicate. To correct the data for photobleaching, we performed a control experiment to quantify the effect of photobleaching and calculated a correction factor to apply to the experimental data during quantification (Supplementary Fig. 6).

As expected, treating the biofilms for a second time with either DNase I or buffer did not result in significant removal of eDNA (Fig. 7B, C). This confirmed that the patches of recalcitrant eDNA could not be removed by extending the incubation with DNase I. The removal of recalcitrant eDNA was apparent in biofilms treated by micrococcal nuclease, DNase A, or DNase I combined with chloroquine (Fig. 7D–F). The result from micrococcal nuclease treatment was highly variable and therefore not statistically significant, but treatment with DNase A or DNase I and chloroquine removed recalcitrant eDNA with high statistical significance (Fig. 7G). The result is consistent with the properties of Z-DNA, which becomes sensitive to DNase I if treated with chloroquine, and we show for the first time that an experimental DNase, DNase A, can degrade non-canonical DNA structures in vitro and in situ in biofilms.

### *S. mutans* biofilm acidity is unchanged by enzyme treatment

**Fig. 8:**
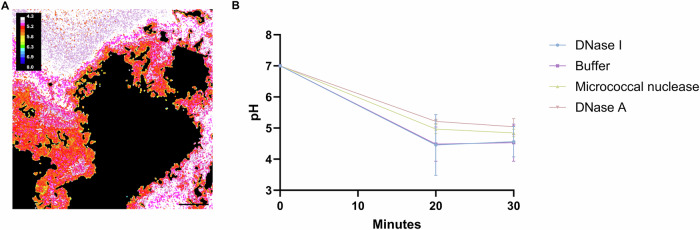
Analysis of biofilm acidity in response to enzyme treatment. *S. mutans* aggregate biofilms were labelled with C-SNARF-4 and the biofilms acidified their surroundings after exposure to sucrose. Images were recorded after 20 and 30 min, and the pH was calculated as the ratio of pixel values in the medium surrounding the biofilm in the green and red channels. **A** False colouring to represent biofilm pH. **B** The results showed that all biofilms were highly acidic, and this was unchanged by treatment with enzymes. The pH in all samples started at 7 and dropped in response to exposure to sucrose irrespective of whether biofilms were treated with enzymes prior to the measurement. Experiments were performed in triplicate with 5 FOV recorded per biological replicate.

DNase treatment removed eDNA from *S. mutans* biofilms, but it did not break up the biofilm structure. However, we wondered if the change in biofilm matrix composition could affect the pathology of oral biofilms by affecting the accumulation of acids in the biofilm matrix during exposure to sucrose. Caries is caused by the local acidic pH caused by fermentation of sugars in the biofilm^34,35^. Kim et al. showed that compact biofilm structures formed by *S. mutans* surrounded by a ring of *S. oralis* produced acid which was responsible for demineralising enamel and causing caries^34^. The *S. mutans* aggregate biofilms in our study have a similarly compact architecture, and we were interested in investigating whether these biofilms were also acidic. eDNA is negatively charged and could interact with positively charged ions in the biofilm environment. We therefore hypothesised that eDNA might act as a proton trap, and that removal of eDNA by enzymes would release trapped protons and increase the pH, which is potentially beneficial for the treatment of caries. We investigated the effect of treatment with DNase I, micrococcal nuclease, the experimental enzyme DNase A, and buffer on biofilm pH using confocal microscopy-based pH ratiometry with C-SNARF-4, a pH sensitive dye that changes emission spectra based on local pH values (Fig. 8A). We exposed treated and untreated *S. mutans* biofilms to sucrose to trigger acid production by the biofilm, then washed the biofilms to remove the sucrose, and used C-SNARF-4 to measure how much the biofilms acidified the environment immediately surrounding the biofilm after 20 and 30 min. We expected that untreated biofilms (rich in eDNA) would be more acidic than enzyme-treated biofilms (less eDNA) and would remain acidic after 20 and 30 min of exposure to sucrose.

The *S. mutans* biofilms were all highly acidogenic, regardless of whether they were treated by enzymes to remove the eDNA, and biofilm acidity did not change during the 30 min window of measurement for any of the conditions tested (Fig. 8B). This demonstrates that the *S. mutans* aggregate biofilm model has an acidogenic phenotype that could be associated with caries but it gives no indication that removing eDNA alters the biofilm pH.

## Discussion

eDNA is an important component of the extracellular matrix of dental biofilms^1,19,20^. Our research supports this and furthermore demonstrates that a fraction of eDNA in dental biofilms is in the Z-DNA conformation. For the first time, we show that dental biofilms also contain G4 structures (Figs. 1–4). In general, these structures comprised a relatively small fraction of the total dental biofilm in both healthy and caries-active subjects, but they were present in the majority of samples tested from both groups. Secondary eDNA structures filled the extracellular matrix of dense bacterial clusters (Figs. 1B, C, 2B, C, 3B, C, 4B, C), and were sometimes also associated to the surface of individual cells (Figs. 1F, 2E, F, 3F, 4F). This indicates that local conditions promote the formation of secondary DNA structures, and that particular species within the diverse microbial community of dental biofilms have the ability to form and associate with such DNA structures.

eDNA in dental biofilms originates both from bacterial cells and from the host^36^, and previous research shows that bacteria manipulate both bacterial and host eDNA to adopt the Z-DNA conformation^30,33^. Local hot-spots with high concentrations of eDNA in general, and Z-DNA in particular, therefore depend on a number of potential factors. Bacteria acquire eDNA from neighbouring cells through processes of autolysis or allolysis, and eDNA from host cells arrives when neutrophils expel their chromatin as neutrophil extracellular traps (NETs). The conversion of eDNA from the B- to Z-DNA conformation then requires additional local factors, such as DNABII proteins released from bacterial cells. Our analysis of dental plaque was purely qualitative, but further analysis into the local species composition, matrix composition, and presence of e.g. histones associated with NETs could point to the origin of Z-DNA, the local conditions, and the microorganisms responsible for hotspots of Z-DNA and G4 structures in the biofilm. We did not include such analysis in the current study because fluorescence immunolabeling must be conducted on fresh samples to preserve the DNA structures, while fluorescence in situ hybridisation to identify the bacteria in question must be conducted on fixed samples. Future in-depth investigations of this kind will no doubt improve our understanding of how these DNase I-resistant structures form in dental biofilms.

Our results highlight that G4 and Z-DNA structures are present in dental biofilms from the majority of subjects, and we find no indication that healthy and caries-active subjects differ in this respect. We collected plaque from the buccal surface, which is easily reached during toothbrushing. We therefore expect that the dental biofilms were relatively young (6–12 h), and the abundance of eDNA and secondary structures may change if biofilms are left undisturbed for longer periods of time. For example, Buzzo et al.^30^ showed in pure culture biofilms that Z-DNA increased in abundance the longer biofilms were left to grow. It is therefore noteworthy that they are present in the majority of dental biofilms sampled in this study.

The high concentration of Z-DNA and G4 structures in large bacterial clusters shows that non-canonical DNA structures can dominate the matrix composition locally. This is important to notice because even local patches of highly resilient biofilm can affect oral health. Wen et al.^33^ showed how the resilience of Z-DNA led to long-term build-up of eDNA, causing calculus formation. Our discovery of Z-DNA and G4 hotspots in dental biofilms raises new questions about how these structures affect biofilm resilience and oral health. It is not always the biofilm at large, but rather sub-structures with a specific phenotype that cause oral disease. Kim et al.^34^ showed that bacterial clusters of *S. mutans* surrounded by a rim of *S. oralis* were responsible for acid production and enamel dissolution leading to enamel demineralisation. The two populations were distinctly segregated in a core-shell structure connected via a scaffold of extracellular matrix produced by *S. mutans. S. mutans* produces eDNA in the biofilm matrix, and in vitro analyses reveal Z-DNA in pure culture biofilms of the species^30^. Having observed several bacterial clusters with a core-shell structure of Z-DNA/B-DNA in dental biofilms, we propose to pursue investigations of how non-canonical DNA structures might contribute to more virulent sub-phenotypes within dental biofilms.

The antibody used for detecting G4 in our study binds to both DNA and RNA G4, and likewise, the antibody used to detect Z-DNA also binds Z-RNA. Extracellular RNA has recently been shown to be an important component of some biofilms^37,38^ and therefore it is possible that dental biofilms contain both G4 DNA and RNA, and both Z-DNA and Z-RNA. We expected that G4 DNA would only form in regions that colocalise with B-DNA, yet in some cases G4 were detected in regions that lacked B-DNA (Figs. 1D, 3B), which may suggest that some of the G4 structures were comprised of RNA and not DNA.

G4 and Z-DNA structures resist degradation by mammalian DNase I, which has been shown in a number of biofilms, including Z-DNA in dental calculus^29,30,33^. Our results confirm the trend, showing that G4 and Z-DNA remain in dental biofilms after treatment with DNase I (Fig. 5). To our surprise, B-DNA associated with these structures also remained in the biofilm after DNase treatment, indicating that DNase I cannot degrade some of the B-DNA within DNA superstructures that contain non-canonical DNA.

To further investigate enzyme removal of secondary eDNA structures, we developed an *S. mutans* biofilm model that is rich in eDNA and contains G4 and Z-DNA structures, and applied this model for studying enzymatic removal of eDNA that cannot be removed by DNase I. It was surprisingly difficult to develop an *S. mutans* model that was rich in eDNA and secondary eDNA structures, despite numerous reports in the literature that eDNA is a component of *S. mutans* biofilms and one report that *S. mutans* biofilms contain Z-DNA^22,30,39–43^. There was a striking difference in the quantity of eDNA present in *S. mutans* biofilms when grown in microwells (Supplementary Fig. 4) compared to when grown as aggregate biofilms suspended in a flask: the quantity of eDNA dramatically increased in aggregate biofilms grown in flasks, as did the quantity of G4 and Z-DNA (Fig. 6). This is possibly due to increased mechanical stress and oxidative stress imposed on the suspended aggregates compared to solid/liquid interface biofilms, as these factors promote formation of the secondary DNA structures^29^.

We identified two treatments that were highly effective at removing eDNA that was recalcitrant to removal by DNase I (Fig. 7). Firstly, an experimental nuclease (DNase A) that had activity against G4 and Z-DNA oligos in vitro was capable of removing much of the recalcitrant eDNA on its own, and secondly, DNase I treatment combined with chloroquine made the previously recalcitrant eDNA susceptible to DNase I. Chloroquine is a DNA intercalator that converts Z-DNA into B-DNA and can be combined with DNase I to remove Z-DNA in biofilms^30^. Together, our results strongly suggest that the patches of recalcitrant eDNA seen in *S. mutans* aggregate biofilms that persisted after treatment with DNase I do indeed contain secondary eDNA structures that are sensitive to DNase A and that can be destabilised by chloroquine.

However, micrococcal nuclease was unable to remove recalcitrant eDNA as effectively as DNase A despite having documented activity against G4 and Z-DNA oligos in vitro (Table 1). Micrococcal nuclease has even been shown to remove G4 in other biofilms^29^. In some FOV, we observed that micrococcal nuclease was highly effective at removing recalcitrant eDNA, but this did not occur frequently enough for the data to be statistically significant (Fig. 7). The large degree of variation in how well the two different enzymes removed recalcitrant eDNA might indicate that eDNA exists in numerous secondary conformations that are not affected equally by both enzymes. Access of the enzymes to the biofilm may have also been hindered by other matrix components. Polysaccharides and eDNA can colocalise in *S. mutans* biofilms^39,44,45^, facilitated by electrostatic interactions similar to what is seen in *Pseudomonas aeruginosa* biofilms^46^. Furthermore, dietary sucrose and starch, which increase the production of polysaccharides in *S. mutans* biofilms, also increase eDNA production which suggests that the two matrix components might interact with one another^47^. eDNA triggers the formation of acidic biofilms in *P. aeruginosa*, and low pH enhances binding between eDNA and polysaccharides in *Myxococcus xanthus* biofilms^48,49^. Interaction between eDNA and polysaccharides might therefore be especially important in cariogenic biofilms which are acidogenic.

This indicates that the best strategy for controlling dental biofilms with enzymes is the combination of different enzymes in formulations that target several matrix components simultaneously. Mutanases and dextranases that target different polysaccharides can remove much, but not all, of in vitro-grown oral biofilms^13,14^. Polysaccharides dominate these in vitro-grown biofilms, but eDNA including Z-DNA and G4 is an important component of real in vivo-grown dental biofilms which originates from bacteria and the host^33,36^. Incorporating nucleases with activity against G4 and Z-DNA in biofilms into these formulations may therefore be the next important step in developing improved products for controlling oral biofilms. While chloroquine would be unsuitable for using in the oral cavity, our results also indicate that developing innovative adjuvant treatments that convert secondary eDNA structures into the DNase I-sensitive B-DNA form would greatly improve the efficacy of enzyme formulations as well.

## Methods

### Materials, bacterial strains, and growth conditions

For fluorescence microscopy experiments, immunolabelling was used to visualise G4, Z-DNA, and B-DNA using a modified version of the protocol from Minero et al.^29^. BG4 goat IgG with Atto-488 conjugation (Ab00174-24.1, Absolute Antibody) was used to visualise G4 structures, and Z22 rabbit IgG with Atto-488 conjugation (Ab00783-23.0, Absolute Antibody) was used to visualise Z-DNA. The fluorophore was the same and therefore G4 and Z-DNA were visualised independently. B-DNA was visualised simultaneously with either G4 or Z-DNA using a primary Anti-dsDNA mouse IgG (ab27156, Abcam) paired with a secondary goat anti-mouse IgG with Alexa Fluor 405 Plus conjugation (A48255, Invitrogen). The primary BG4, Z22, and Anti-dsDNA antibodies were diluted 1/100 in blocking buffer (3% bovine serum albumin (BSA; A7906, Sigma-Aldrich) in 1 x phosphate buffered saline (PBS; E703, VWR)), and the secondary antibody was diluted 1/150 in blocking buffer. Microorganisms were visualised using the membrane stain FM 4-64 (10 µg/mL in 1 x PBS; T13320, Invitrogen).

*S. mutans* DSM 20523 was cultured on brain heart infusion (BHI; 53286, Millipore) agar (05040, Millipore) for 72 h and grown aerobically in BHI medium until late exponential phase prior to experimental use. Biofilms were grown in BHI medium supplemented with 1 % sucrose (57903, Sigma Aldrich) and 200 mM NaCl (S5886, Sigma Aldrich). eDNA was visualised in biofilms by incubation with 0.5 μM SYTOX Green Nucleic Acid Stain (S7020, Invitrogen) diluted in 1 x PBS.

The following commercial enzymes were used in this study at the following concentrations: 500 U/mL DNase I (04716728001, Roche) and 15 U/mL micrococcal nuclease (EN0181, Thermo Scientific). DNase I solutions were supplemented with 5 μM chloroquine (J64459.14, Thermo Scientific) where relevant. One experimental enzyme was also used (500 ppm DNase A), produced by Novonesis A/S. All enzymes were diluted in a universal buffer optimised for enzyme activity comprising 25 mM Tris-HCl (RES3098T-B7, Sigma-Aldrich), 6.25 mM CaCl_2_ (223506, Sigma-Aldrich), 1 mM MgSO_4_ (M1880, Sigma-Aldrich), pH 7.5.

### Collection of dental biofilms

Supragingival plaque was collected from 10 healthy volunteers with no clinical signs of periodontal disease or active caries lesions, and from 10 caries-active patients with at least three active caries lesions who were undergoing treatment for caries at the Department of Dentistry and Oral Health, Aarhus University. The Nyvad criteria and the periodontal screening index were used to assess the caries and periodontal status, respectively^50,51^. Plaque from caries-active patients was collected from both cavitated and non-cavitated active caries lesions. Samples were collected between October 2023 and May 2024. Pooled plaque was collected from all buccal tooth surfaces of the first quadrant and transferred to a sterile Eppendorf tube. Sterile curettes were used for sampling; the three-dimensional plaque architecture was therefore compromised to some extent. Plaque was used for experiments immediately after collection. All volunteers signed informed consent forms. The protocol was approved by the Ethical Committee of Region Midtjylland (case no. 1-10-72-178-18).

### Development of eDNA-rich in vitro oral biofilm model for investigation of DNase activity in biofilms

We sought to develop an oral biofilm model rich in eDNA, G4, and Z-DNA to enable robust quantification of DNase activity against these recalcitrant structures in biofilms. We grew biofilms inoculated with plaque (pooled from 9 healthy volunteers, mixed with glycerol (25 %) and stored at −80 °C until use) or with single species of oral bacteria (of *Lactobacillus paracasei, Lactobacillus rhamnosus, Fusobacterium nucleatum,* *Porphyromonas gingivalis* *Prevotella intermedia, Streptococcus mitis, Streptococcus oralis, Streptococcus gordonii,* and *Streptococcus mutans*). We tested a variety of media (BHI (53286, Millipore), tryptic soy broth (TSB; T8907, Sigma Aldrich), or plaque medium^52^), and modified the media and conditions to match those previously reported to affect formation of eDNA and non-canonical DNA structures (Supplementary Table 1). Supplements to the biofilm media included NaCl (S5886, Sigma Aldrich), CaCl_2_ (102382, Merck), KCl (P9333, Sigma Aldrich), sucrose (57903, Sigma Aldrich), fetal bovine serum (FBS, A3160802, Thermo Fisher Scientific), chloramphenicol (Cm, C0378, Sigma-Aldrich), hemin (51280, Merck), yeast extract (Y1625, Sigma Aldrich), and a synthetic competence-stimulating peptide (CSP)^27^. Biofilms were inoculated into 96 well plates (89621, ibidi) and incubated at 37 °C with medium exchange every 24 h before imaging with confocal microscopy. Biofilms were stained with 0.5 µM SYTOX Green or 2 μM TOTO-3 (eDNA) and 10 μg/mL FM 4-64 (cells) to assess the amount of eDNA in general, or by immunolabelling to visualise the different eDNA structures (G4, Z-DNA and B-DNA).

### Preparation of *S. mutans* aggregate biofilms

The biofilm model with the most reproducible production of eDNA was *S. mutans* suspended aggregate biofilms, and these were used for subsequent analyses of DNase treatment. Freshly grown *S. mutans* colonies were scraped from an agar plate and inoculated into 20 mL BHI supplemented with 1 % sucrose and 200 mM NaCl in an Erlenmeyer flask. Flasks were incubated overnight (16–18 h) at 37 °C with 150 rpm shaking. *S. mutans* formed small aggregate biofilms suspended in the medium which did not have any noticeable planktonic growth.

### Visualisation of eDNA, G4, Z-DNA, and B-DNA in biofilms

To visualise G4, Z-DNA, and B-DNA, confocal laser scanning microscopy (CLSM) of fluorescently conjugated antibodies specific to these DNA structures was used. All immunolabelling was performed on live, fresh dental biofilms that were processed and imaged immediately after collection, or on *S. mutans* aggregate biofilms grown in the laboratory. The labelling protocol was modified from Minero et al.^29^, all steps were performed at room temperature, and all incubations were performed in the dark.

To label dental biofilms, a hydrophobic marker was used to draw a small ring on positively charged microscope slides (Superfrost, 631-0108, VWR). Each dental biofilm sample was split into two, and each half was transferred to separate microscope slides to visualise G4 and Z-DNA in the same sample. Samples were first blocked with 60 μL blocking buffer for 30 min, then hybridised with 60 µL BG4 or Z22 antibody in blocking buffer for 1 h before the addition of 60 μL anti-dsDNA antibody and further incubated for 1 h. The samples were washed by removing the liquid and replacing it with 120 μL 1 x PBS twice before addition of 60 μL Alexa Fluor 405 Plus-conjugated secondary antibody and incubation for 1 h to label the anti-dsDNA primary antibody. The liquid was then discarded, and samples were washed twice with 120 µL 1 x PBS. Finally, 30 µL FM 4-64 was added and incubated in the dark for 5 min. A microscope coverslip was placed on top of the samples and the edges were sealed with nail polish. The samples were imaged immediately by CLSM (LSM700, Zeiss).

To label *S. mutans*, aggregate biofilms (200 µl volume) were transferred to Lo-Bind Eppendorf tubes (EPPE0030108.418, VWR) and immunolabelling was performed in these tubes using 100 μL volumes instead of 60 μL. The incubation time with the BG4/Z22 antibodies was increased to 3–4 h and B-DNA labelling was omitted. In separate experiments, cells and eDNA were visualised by staining biofilms with 200 µL staining solution (0.5 µM SYTOX Green and 10 µg/mL FM 4-64) and incubating for at least 15 min prior to visualising with CLSM (LSM700, Zeiss).

CLSM imaging was performed using a Plan-Apochromat 63x/1.40 NA oil immersion objective lens. eDNA (SYTOX Green) and cells (FM 4-64) were visualised using a 488 nm 10 mW laser operating at 2 % power. Emissions were split into separate imaging channels using a filter to collect fluorescence emissions above and below 644 nm. To image B-DNA (Alexa Fluor Plus 405), G4 (Atto 488), and Z-DNA (Atto-488), 5 mW 405 nm and 10 mW 488 nm lasers operating at 3 % and 4.5 % powers were used. For imaging dental biofilms, the samples were highly variable, and thus fields of view (FOV) were chosen to show locations of the plaque that contained G4 or Z-DNA, and notes were kept to qualitatively describe the appearance of the biofilms. A minimum of 3 FOV were imaged per sample, which were a mixture of Z-stacks and 2D snapshots. Imaging settings were varied due to variations in fluorescence intensity in different biofilm locations by adjusting the master gain and pixel dwell time. Image brightnesses in the figures were therefore adjusted individually in Fiji ImageJ^53^ for presentation purposes, and the data is descriptive of whether different eDNA structures were present in the samples and are not quantitative. For imaging *S. mutans* biofilms, the FOV was arbitrarily chosen to assess the presence of eDNA, G4, and Z-DNA. All *S. mutans* experiments were performed with at least 3 biological replicates, and sample preparation and imaging settings were kept identical in quantitative experiments.

### Verifying DNase I activity

To verify DNase I activity, we treated salmon sperm DNA (100 μg, D1626, Sigma-Aldrich) for 2 h at 37 °C with DNase I (500 U/mL). 100 ng of the DNase I-treated and an untreated control were loaded onto an agarose gel (1% agarose in 0.5 X TBE buffer) alongside a ladder (TrackIt 1 kb Plus DNA Ladder, 10488085, Invitrogen), and gel electrophoresis was performed at 120 V for 1.5 h before staining with SYBR Safe (S33102, Invitrogen) and visualisation in a Gel Doc EZ Imager (BioRad) with Image Lab Software (Biorad) (Supplementary Fig. 1).

### DNase I treatment of dental biofilms

Supragingival plaque was collected from one subject with no clinical signs of periodontal disease or active caries lesions, whose plaque contained G4 and Z-DNA structures. The sample was distributed into multiple tubes and suspended in 100 µL PBS. The PBS was removed and replaced by either 200 µL DNase I (500 U/mL) diluted in buffer (25 mM Tris-HCl, 6.25 mM CaCl_2_, 1 mM MgSO_4_, pH 7.5) or 200 µL buffer with no enzyme. Samples were incubated for 1 h at 37 °C, followed by immunolabelling to visualise G4, Z-DNA, B-DNA, and microorganisms as described above. Immunolabelling was performed in tubes rather than on microscope slides. Finally, the labelled samples were transferred to microscope slides and visualised by CLSM (LSM700, Zeiss) using a Plan-Apochromat 63x/1.40 NA oil immersion objective lens with 5 mW 405 nm and 10 mW 488 nm lasers operating at 3 % and 4.5 % power, respectively. The imaging settings for visualising G4, Z-DNA, and B-DNA were kept the same when imaging samples with and without DNase I treatment, so that the conditions could be compared. At least 5 FOV were acquired per sample and the experiment was performed in triplicate on separate days.

### Screening nuclease activity towards synthetic G4 and Z-DNA substrates

DNA molecules were purchased from Integrated DNA Technologies (IDT) as stocks of 100 µM in 1 mM TE buffer (pH 7.5). All salts as well as chitosan (high purity, 74006) were purchased from Merck. Oligos and buffers are listed in Table 2. Solutions of 1 µM DNA were incubated at 95 °C for 5 min and gradually cooled down to 35 °C over 90 min to anneal and fold into G4 or Z-DNA structures. The G4 and Z-DNA conformations were validated using circular dichroism (Supplementary Fig. 7).

**Table 2:** ssDNA sequences and their corresponding buffers used to prepare and treat B-DNA, Z-DNA, and G4-DNA with different nuclases

Structure	Sequence (5’-3’)	Buffer (folding DNA)	Buffer (enzyme treatment)
B-DNA (oligo B1-B1c)	GTG GCA GGT CAG TCA AGT ATA CTG CAC TA	25 mM Tris-HCl, 6.25 mM CaCl2, 1 mM MgSO4, pH 6	30 mM Tris-HCl, 6.25 mM CaCl2, 1 mM MgSO4, pH 6
Z-DNA (oligo B1-B1c)	GTG GCA GGT CAG TCA AGT ATA CTG CAC TA	25 mM Tris-HCl, 6.25 mM CaCl2, 1 mM MgSO4, 0.025% chitosan, pH 6	30 mM Tris-HCl, 6.25 mM CaCl2, 1 mM MgSO4, pH 6
Z-DNA (oligo B2-B2c)	GCG CGC GCG CGC GCG CGC GCG C	25 mM Tris-HCl, 6.25 mM CaCl2, 1 mM MgSO4, 0.025% chitosan, pH 6	30 mM Tris-HCl, 6.25 mM CaCl2, 1 mM MgSO4, pH 6
G4-DNA (oligo Tel)	TTA GGG TTA GGG TTA GGG TTA GGG TTA	25 mM Tris, 100 mM KCl, pH 7	30 mM Tris, 20 mM KCl, 6.25 mM CaCl2, 1 mM MgSO4, pH 6
G4-DNA (oligo C-myc)	GAG GGT GGG TAG GGT GGG	10 mM Tris, 100 mM KCl, pH 7	27 mM Tris, 20 mM KCl, 6.25 mM CaCl2, 1 mM MgSO4, pH 6

Double-stranded B-DNA and Z-DNA were prepared by mixing the oligo with its complementary strand, while G4-DNA was folded from the ssDNA oligo.

For commercial nucleases with defined activity, 4 U/mL of DNase I and 0.12 U/mL micrococcal nuclease were used. For the experimental nuclease, a standard concentration of 10 mg/L was used.

The buffer, DNA, and enzymes were assembled directly in Nunclon Delta Surface black bottom 96-well plates (Thermofisher Scientific 137101). As a negative control, Milli-Q water was added instead of enzymes. 10 wells were filled with pure 1x buffer. The plate was sealed and incubated at 37 °C at 50 rpm for 1 h. To quantify the DNA fractions remaining after enzymatic treatments, a DNA-binding dye was added to all wells. 1 µM SYTO60 (Thermofisher) was used for G4 and 0.5 % Picogreen (Thermofisher) for Z-DNA. The fluorescence of the dyes was measured in a plate reader (CLARIOstar) using the following settings: (i) 630-10 nm excitation/ 670-10 nm emission (gain 2500) for G4, and (ii) 488-15 nm excitation/ 530-20 nm emission (gain 1800) for Z-DNA. The averaged autofluorescence of the dyes in the buffer was subtracted. Then, the fluorescence values across different replicates (at least 3) without DNase were averaged. Finally, the fluorescence values from DNA treated with a particular enzyme was divided by the fluorescence value of the no enzyme control to obtain the “DNA fraction remaining”. These fractions in different replicates (at least 3) were averaged individually for each DNA structure

### *S. mutans* aggregate biofilm preparation for enzyme treatment combined with CLSM

Liquid containing 500 μL aggregate biofilms was transferred to a sterile Lo-Bind Eppendorf tube. The supernatant was carefully discarded and replaced by 200 μL PBS to wash the biofilms. The supernatant was discarded again and replaced by 200 μL staining solution (0.5 μM SYTOX Green and 10 μg/mL FM 4-64 in 1 x PBS) and incubated in the dark for 30 min. Meanwhile, a sandwich chamber was prepared by affixing a 65 μL gene frame (AB0577, Thermo Scientific) to a microscope glass slide and using a sterile scalpel to cut and inlet and outlet. 65 μL fluorescently stained *S. mutans* aggregate biofilms were transferred to the gene frame and secured with an 18 ×18 mm microscope coverslip (Fig. 6B). The samples were washed with 200 μL PBS by pipetting the solution into the sandwich chamber inlet whilst using tissue to wick away the staining solution from the outlet.

### Time-lapse CLSM imaging of eDNA removal in *S. mutans* aggregate biofilms

The staining solution was removed and replaced by 200 μL DNase I (500 U/mL in buffer). The sandwich chamber inlet and outlet were sealed using dental silicone to prevent evaporation of the enzyme solution. The sample was transferred to a CLSM stage top incubator (H301K, Okolab) and incubated for 2 h at 37 °C whilst performing time-lapse imaging. The same CLSM imaging settings for eDNA (SYTOX Green) and cells (FM 4-64) as described above were used and Z-stacks (5.3 µm height) were acquired once every 2 min for a total time of 2 h.

### Enzyme treatment of recalcitrant eDNA in *S. mutans* aggregate biofilms

In subsequent experiments, after staining and mounting biofilms in the sandwich chamber, the biofilms were pretreated with DNase I without visualising with CLSM. DNase I (200 µL) was flowed through the sandwich chamber, which was then transferred to a humidity chamber and incubated in the dark for 2 h at 37 °C. The biofilms were washed with 200 µL PBS, and then imaged by CLSM using the same settings for eDNA (SYTOX Green) and cells (FM 4-64) as described above. Patches of recalcitrant eDNA that were not removed during the DNase I pretreatment were identified and their positions saved in the microscope software. 12-Bit Z-stacks (20 µm) were acquired of 5 FOV containing recalcitrant eDNA (“before” images). The samples were then removed from the CLSM, and 200 µL of different enzyme solutions was flowed through the sandwich chamber. Enzymes tested were: DNase I with chloroquine, micrococcal nuclease, DNase A, DNase I alone, and a control with only buffer (25 mM Tris-HCl, 6.25 mM CaCl_2_, 1 mM MgSO_4_, pH 7.5). Then the sample was incubated in the dark in a humidity chamber for 1 h at 37 °C. Afterwards, the sample was returned to the CLSM and the same 5 FOV were imaged using identical acquisition settings (“after” images). The experiments were performed in biological triplicate.

### Computational analysis of eDNA removal in *S. mutans* aggregate biofilms

CLSM images were quantified to compare how well the different enzymes tested could remove recalcitrant eDNA in *S. mutans* aggregate biofilms. We quantified how the area coverage of eDNA changed in response to the different enzyme and control treatments in single slices of the images. The calculations were restricted to the area of the image that contained the patch of recalcitrant eDNA to lessen the contribution of dead cells to the quantification. To take into account photobleaching and any effect from washing the biofilm introduced when adding the second enzyme, an additional control experiment was performed to determine a correction factor to apply to the calculation (Supplementary Fig. 6).

The “before” and “after” images were aligned using a rigid body transformation in MATLAB to correct small spatial shifts between the images. The red channel (cells) did not change due to enzyme treatment and was therefore used to calculate the transformation matrix required to align the “after” images onto the “before” images. The transformation matrix was then applied to the “after” image to align the green channels (eDNA). All subsequent processing was applied to the green channel images only, using the original “before” image and the aligned “after” image. All subsequent processing was performed using macros written in Fiji ImageJ^53^.

The area containing the patch of recalcitrant eDNA was identified using the “before” image. To do so, a median filter with a radius of 2 pixels was first applied to the image to reduce noise. A threshold was applied using Huang’s fuzzy thresholding method^54^ and small objects were removed from the resulting binary image using an erosion followed by a dilation using a circle structure element with a radius of 3 pixels. Holes were filled and then the particles were analysed and added to the region of interest (ROI) manager. The largest ROI was selected and used to create a mask. This mask was used to locate the region of recalcitrant eDNA for further quantification. The region was always identified using the “before” image, and the same region was quantified in both the “before” and “after” images.

To quantify how the area coverage of eDNA changed, the mask was first applied to the “before” image. A fixed brightness threshold was then applied to the image and the area coverage of pixels with values above the fixed threshold were quantified only within the area determined by the mask. The “after” image was corrected for photobleaching (Supplementary Fig. 6) and then the process was repeated using the same mask. The percentage of eDNA remaining after the enzyme treatment was calculated by dividing the area coverage of pixels above the threshold in the “after” image by the area coverage in the “before” image. The process was repeated for each FOV from each replicate experiment. Image examples of each processing step are given in the supplementary information (Supplementary Fig. 8).

### Ratiometric pH measurements of *S. mutans* aggregate biofilms

*S. mutans* aggregate biofilms (500 µL) were transferred to Lo-Bind Eppendorf tubes. The supernatant was removed and replaced by 200 µL 0.9 % NaCl (pH 7.0) to wash the biofilms. The supernatant was removed again and replaced by 200 µL enzyme solution and the biofilms were incubated for 2 h at 37 °C. Enzymes tested were: DNase I, micrococcal nuclease, DNase A, and a control with buffer only. The biofilms were washed by removing the supernatant and adding 200 µL 0.9 % NaCl twice. Afterwards, 25 µL liquid containing *S. mutans* biofilm aggregates was transferred to a 96-well plate (89621, ibidi) and the liquid surrounding the biofilms was thoroughly removed using filter strips. SNARF™-4F 5-(and-6)-Carboxylic Acid (C-SNARF-4, 20 μM; S23920, Invitrogen) was mixed with 0.9 % NaCl (pH 7.0) supplemented with 4 % sucrose, and 150 µl was added to the microwells. 5 FOV (2D slices located 10 μm deep into the biofilms) were identified using CLSM, and the same FOV were imaged after 20 min and 30 min of incubation with C-SNARF-4.

### C-SNARF-4 calibration and image acquisition

Calibration was conducted using MES buffer solutions (pH range 4.2–8.0) (M0606, TCI Europe). Each buffer solution (50 µM) was mixed with C-SNARF-4 (20 µM) and images were captured in three randomly selected FOV within a 96-well microplate (89621, ibidi) using an inverted confocal microscope (LSM700, Zeiss) with a Plan-Apochromat 63x/1.40 oil-immersion objective lens. A 10 mW 555 nm laser operating at 2 % power was used for excitation, and fluorescence emissions were detected in two channels from 300–618 nm (green channel) and 618–800 nm (red channel). The imaging parameters were as follows: pinhole size of 1.76 AU (optical slice thickness 1.3 µm), image resolution of 512 × 512 pixels (101.61 × 101.61 µm²), pixel dwell time of 12.61 µs, and an 8-bit resolution.

Equation (1) was derived with the MyCurveFit Data Analysis Tool (My Assays Ltd., Brighton, UK) using the dye calibration data to correlate fluorescence emission ratios to pH values. Here, $R$ represents the ratio of fluorescence intensities recorded in the green and red channels. Images of *S. mutans* aggregates were obtained under identical imaging conditions and C-SNARF-4 concentration as the calibration.

1 $${pH}=0.1858191+\frac{2.966341-0.1858191}{{\left(1+{\left(\frac{R}{67.43645}\right)}^{6.33705}\right)}^{3697238}}$$

### Digital image analysis of ratiometric pH images

For calculation of the pH surrounding the *S. mutans* aggregates, the red and green channel images were exported separately as TIF files to the digital image software Fiji ImageJ^53^. A mean filter (pixel radius 1) was applied to all images to compensate for detector noise, and a green image from each field of view was hereafter segmented with a manually chosen intensity threshold that identified extracellular areas but not bacterial cells as objects. Then the object layer (the selection of extracellular areas) was converted to an ROI and saved. For every FOV, the green channel image was divided by its corresponding red channel image, and the matching ROI was applied. For the ROI, ratios and standard deviations were calculated. Then, the ratios were converted to pH values according to Eq. (1).

## Supplementary information

Supplementary information

Supplementary video

## Data Availability

The datasets generated during the current study are available in the Zenodo repository, 10.5281/zenodo.13843287.
