# Supplementary material for: Dental biofilms contain DNase I-resistant Z-DNA and G-quadruplexes but alternative DNase overcomes this resistance
Source: NPJ Biofilms Microbiomes. 2025 May 19;11:80. doi: 10.1038/s41522-025-00694-x (PMC12089357; doi:10.1038/s41522-025-00694-x)
Supplement: Supplementary file 1 — Supplementary information [file 41522_2025_694_MOESM1_ESM.pdf]

Dental biofilms contain DNase I-resistant Z-DNA and G-quadruplexes but alternative DNase overcomes this resistance

Dominique C. S. Evans[1,3], Mathilde F. Kristensen[2], Gabriel Antonio S. Minero[1], Lorena G. Palmén[3], Inge Knap[3], Manish K. Tiwari[3,*], Sebastian Schlafer[2], Rikke L. Meyer[1,4,*]

[1]Interdisciplinary Nanoscience Centre, Aarhus University, Aarhus, Denmark

[2]Department of Dentistry and Oral Health, Section for Oral Ecology, Cariology, Aarhus University, Aarhus, Denmark

[3]Novonesis A/S, Bagsværd, Denmark

[4]Department of Biology, Aarhus University, Aarhus, Denmark

*Corresponding authors: Rikke L Meyer (rikke.meyer@inano.au.dk) and Manish K. Tiwari (MAKT@novonesis.com)

Supplementary Movie 1 caption

Time-lapse CLSM video showing *S. mutans* (magenta) and eDNA (green) in response to treatment with DNase I at 37 °C. The biofilm was prelabelled with FM 4-64 (cells) and SYTOX Green (eDNA) and mounted in a sealed sandwich chamber inside a heated stage-top incubator. Images were recorded in the same location once every 2 min for a total time of 2 h.

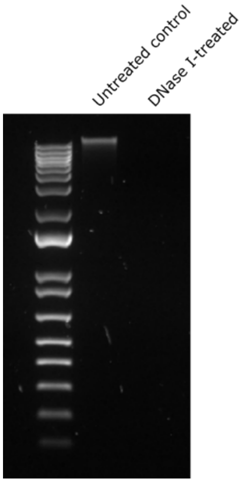

Supplementary Figure 1. Salmon sperm DNA either treated with DNase I (right lane) or untreated (left lane). DNA was treated in buffer for a total time of 2 h at 37 °C. All DNA was removed by DNase I during this incubation.

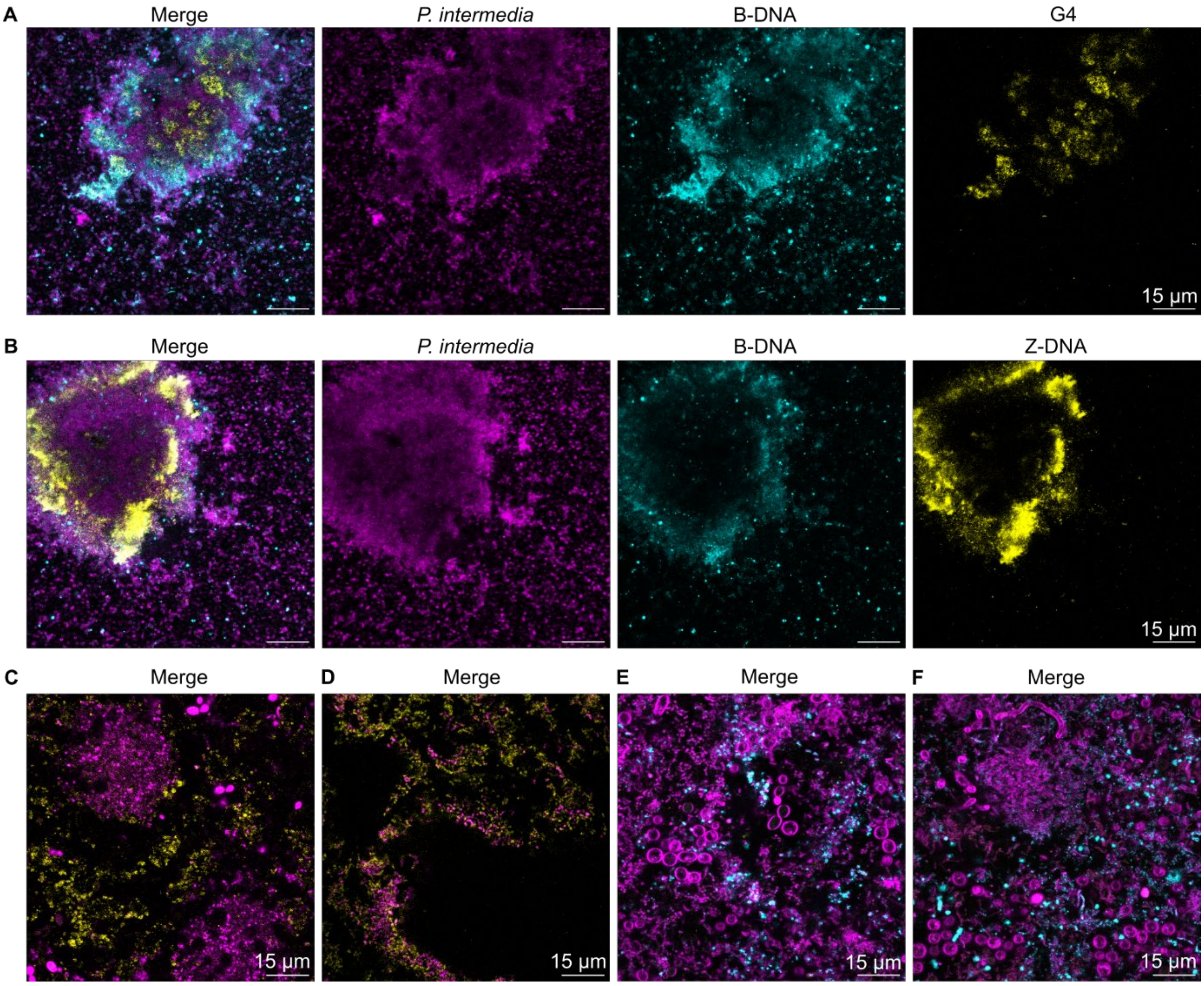

Supplementary Figure 2. Investigations into G4 and Z-DNA formation in *P. intermedia* and mixed species biofilms. *P. intermedia* biofilms (magenta) grown for 48 h in plaque medium supplemented with 1 % sucrose, 100 mM KCl, and 5 µM hemin contained B-DNA (cyan), **A)** G4 (yellow), and **B)** Z-DNA (yellow). Biofilms inoculated with pooled plaque (magenta) in BHI supplemented with 1 % sucrose, 100 mM NaCl, and 5 µM hemin and grown for 7 days aerobically initially contained **C)** G4 and **D**) Z-DNA, but these results were not replicated in a second experiment (**E** = G4 (yellow) and **F** = Z-DNA (yellow)). Cells were visualised using FM 4-64 in panels A-B and E-F and using SYTO 40 in panels C-D. B-DNA, G4, and Z-DNA were visualised by immunolabelling. B-DNA labelling was omitted in panels C-D.

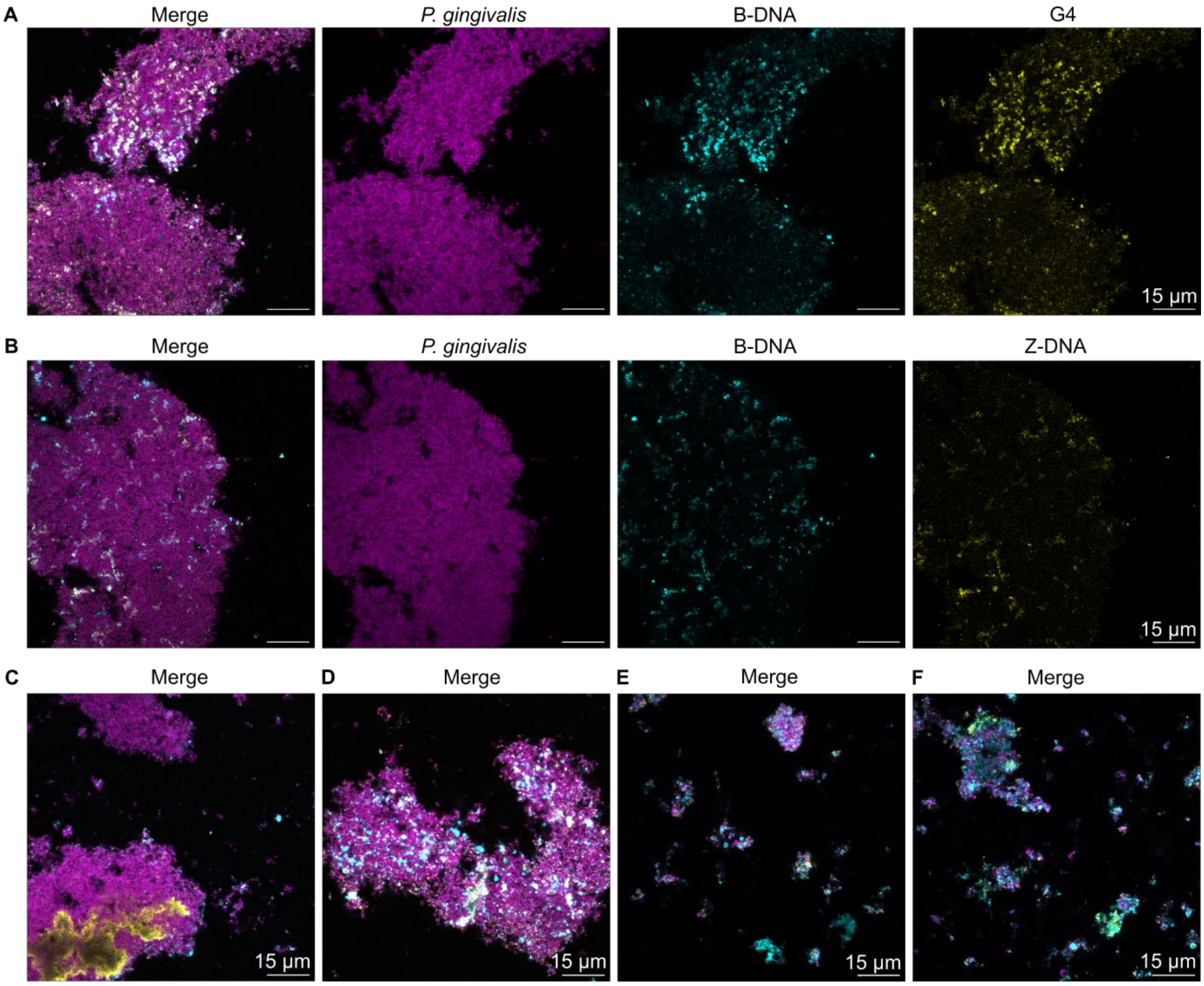

Supplementary Figure 3. Investigations into G4 and Z-DNA formation in *P. gingivalis* biofilms. *P. gingivalis* biofilms (magenta) grown for 48 h in plaque medium supplemented with 1 % sucrose, 100 mM NaCl and 5 µM hemin contained B-DNA (cyan) and **A)** G4 (yellow) and **B)** Z-DNA (yellow). Replacing NaCl with 100 mM KCl also led to **C)** G4 and **D)** Z-DNA formation. **E + F)** Freshly prepared plaque medium resulted in poor biofilm formation compared to the older plaque medium used in panels A-D. Panel E shows G4 and panel F shows Z-DNA. All images show 2D slices of biofilms. Cells were visualised with FM 4-64 and DNA structures with immunolabelling.

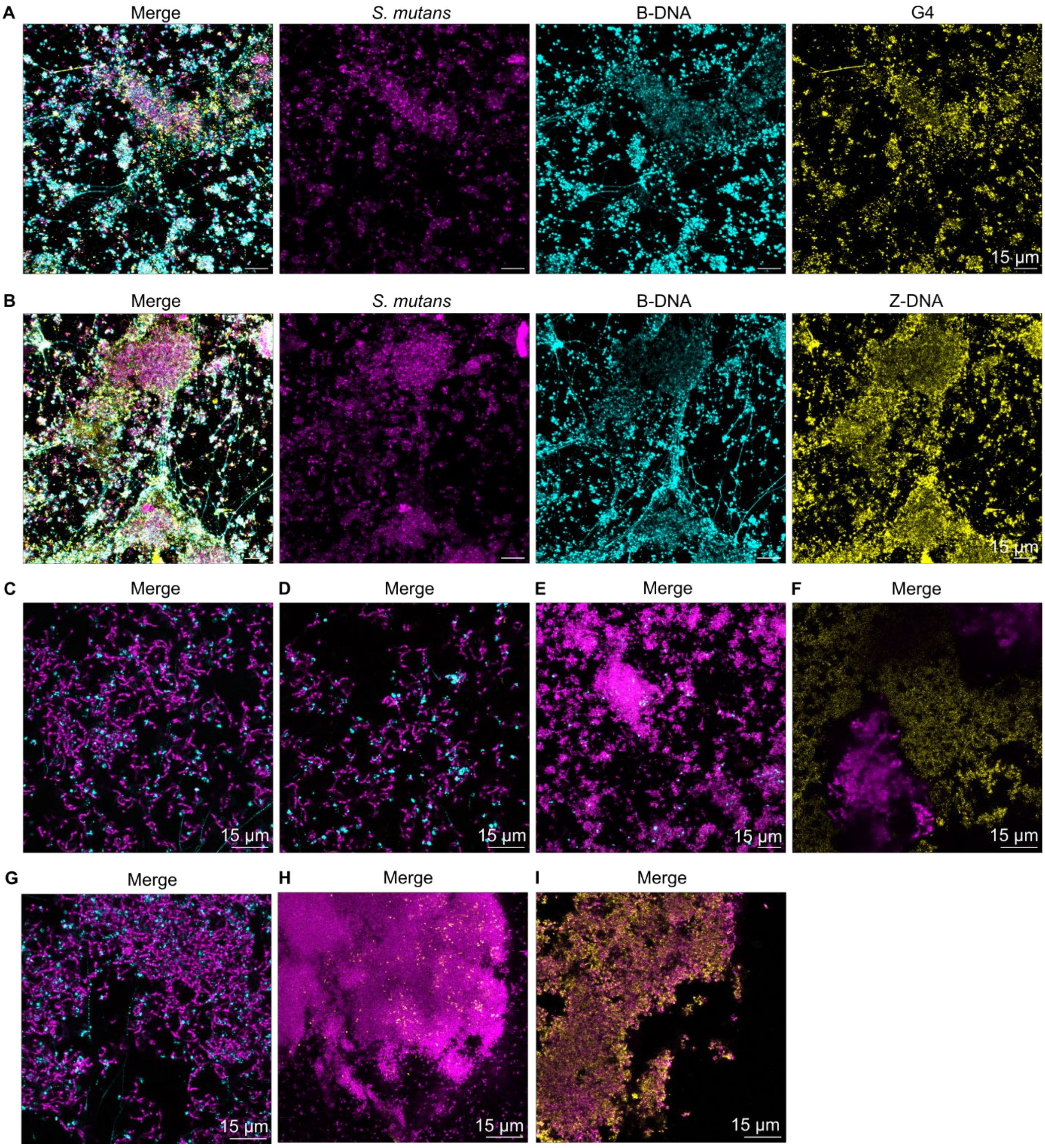

Supplementary Figure 4. Highlights of investigations into G4 and Z-DNA formation in *S. mutans* biofilms. A + B) 48 h *S. mutans* biofilms grown in BHI medium supplemented with 1 % sucrose, 100 mM NaCl and 10 µM hemin initially appeared to contain compacted aggregates of biofilm (magenta), B-DNA (cyan), G4 (yellow) and Z-DNA (yellow). Some later attempts to reproduce these biofilms were unsuccessful as the biofilms did not contain **C)** G4 or **D)** Z-DNA. **E)** Starvation of 24 h old biofilms in sterile saliva for a further 24 h resulted in a large loss of eDNA and G4 compared to the biofilms in panels A and B. **F)** Substituting NaCl for 100 mM KCl and 1 µM CSP initially led to the formation of large G4 structures but **G)** this was not reproduced in later experiments. **H)** Colonies *of S. mutans* scraped from an agar plate contained G4. **I)** Biofilms inoculated from agar rather than planktonic cultures contained G4.

Panels A, B, and E show maximum intensity Z-projections. Panels C-D and F-I show single 2D slices of biofilms. All panels presented show either G4 or Z-DNA labelling. B-DNA labelling was omitted in panels F, H, and I. B-DNA, G4, and Z-DNA were visualised by immunolabelling. Cells were visualised by FM 4-64 in panels A-G and by SYTO 40 in panels H-I.

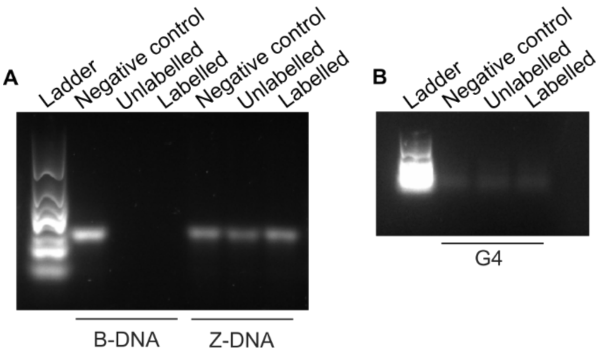

Supplementary Figure 5. Labelling B-DNA, Z-DNA, or G4-DNA oligos with SYTOX Green does not inhibit DNase I. Gel electrophoresis results comparing DNase I digestion results of **A)** B-DNA and Z-DNA, and **B)** G4 oligos with and without SYTOX Green labelling. The negative control was untreated and unlabelled DNA.

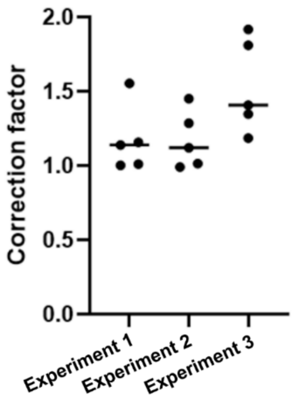

Supplementary Figure 6. Photobleaching correction factors from 3 replicate experiments. Each point represents the correction factor calculated for 1 FOV and black bars represent group medians. Before quantifying how the area covered by eDNA within the masked region changed due to enzyme treatment, a correction factor was needed to account for the effects of photobleaching and washing of the biofilms. To calculate the correction factor, 5 FOV containing recalcitrant eDNA in *S. mutans* biofilms were imaged under identical settings to the original experiments. Afterwards, the biofilms were washed with 200 µl buffer in lieu of adding enzyme, and then the incubation step was skipped and the same 5 FOV were imaged again immediately. Therefore, the fluorescence only changed due to washing and repeat imaging of the same FOV and not due to enzyme activity. The experiment was performed in triplicate and the imaging settings used were identical to the ones used in the original experiments. The patches of recalcitrant eDNA were identified using the computational method described in the main document, and the mean pixel values of the "before" and "after" images were calculated within that region. The ratio of the mean pixel values was used to calculate a correction factor for each FOV. and the mean value across the three triplicate experiments was used. The final correction factor was 1.293159744 ± 0.208526176 (mean ± SD).

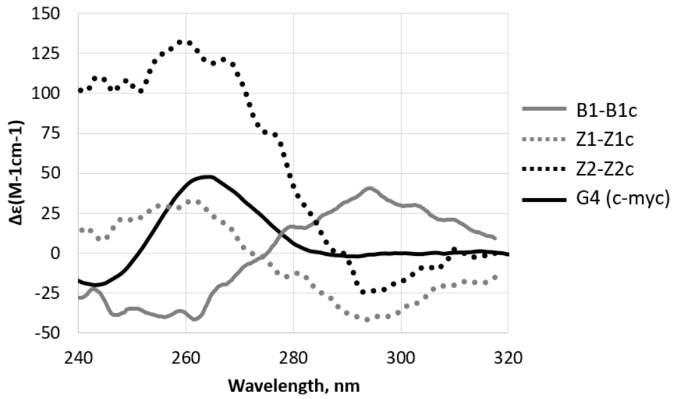

Supplementary Figure 7. Characterization of annealed DNA by Circular Dichroism. B1-B1c represents B-DNA and was annealed in in 25 mM Tris-acetate buffer with 6.25 mM CaCl2, and 1 mM MgSO4 (pH 6). Z1-Z1c had the same oligonucleotide sequence as B1-B1c and Z2-Z2c had self-complementary polyGC sequence. Both were annealed in in 25 mM Tris-acetate buffer with 6.25 mM CaCl2, and 1 mM MgSO4 (pH 6) in the presence of 0.025 % chitosan that was shown to to flip B-DNA into Z-DNA at pH 6. Flipping of B-DNA into Z-form was identified as the positive peak shift from 290 nm to 260 nm and at the same time appearance of a negative peak at 290 nm. Both B- and Z-DNA CD spectra were obtained after 5-fold diluting DNA as well as chitosan (to the working DNA concentration of 200 nM). G4 oligo c-myc was annealed in 25 mM Tris-acetate buffer c-myc, tel, nonGQ and B1-B1c DNA diluted in 25 mM Tris-acetate buffer with 20 mM KCl (pH 6). The parallel G-quadruplex was identified as the positive peak at 260 nm.

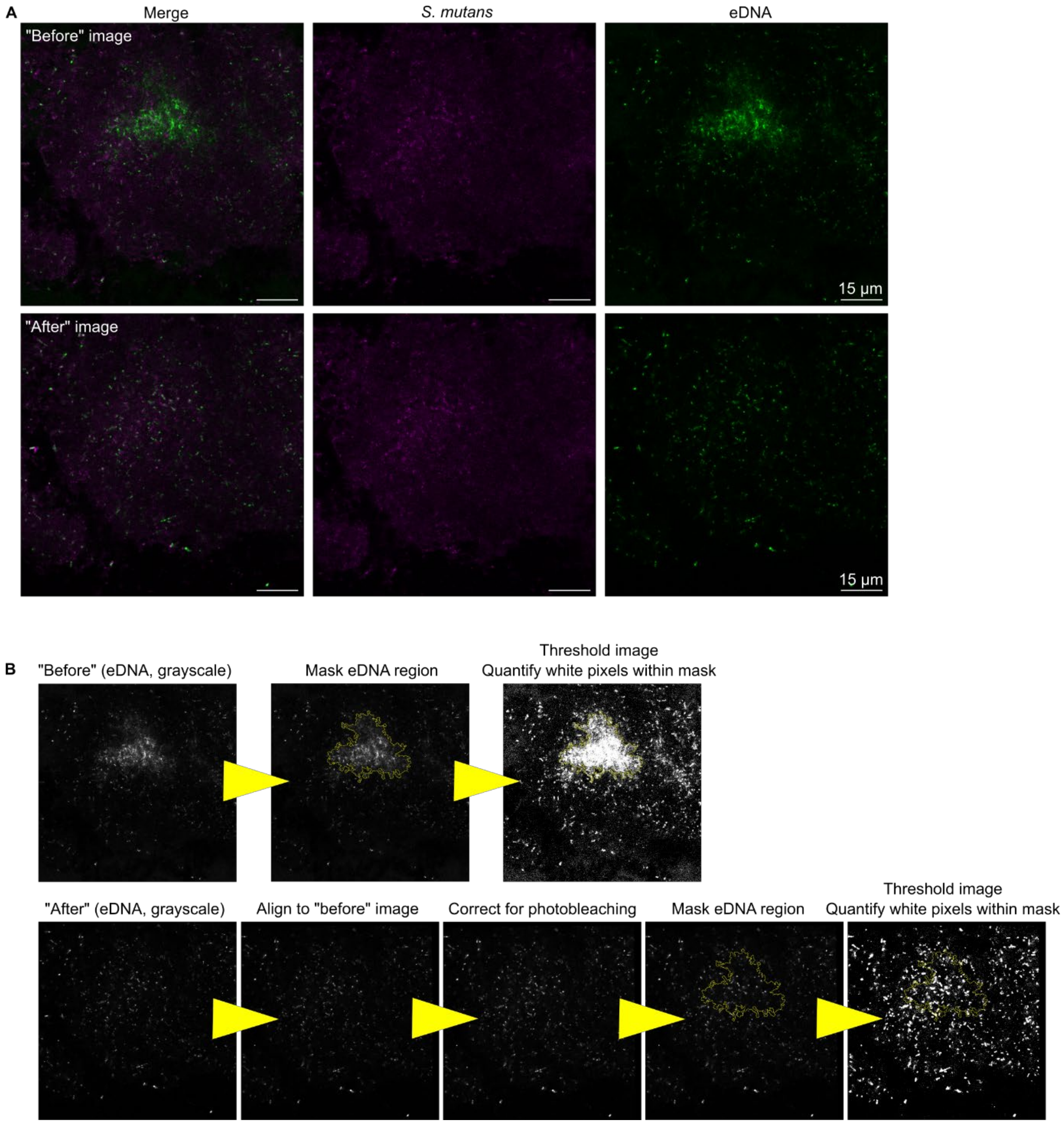

Supplementary Figure 8. Image processing step by step. A) Example of raw data for quantification showing merged and single channels of "before" and "after" images of a sample treated by enzymes. **B)** Grayscale and binary images showing the stages of image processing for the "before" and "after" images.

Supplementary Table 1. List of biofilm growth conditions screened for eDNA, G4, Z-DNA, and/or B-DNA production. M = microaerophilic, AN = anaerobic, AE = aerobic, Y = yes, N = no, W = weak. BHI = brain heart infusion medium, TSB = tryptic soy broth medium, and CSP = competence-stimulating peptide. Samples depicted in Supplementary figures are marked in grey shade.

		**Medium components**									**Initial screening results**					
	**Species**	**Inoculum (OD)**	**Medium**	**Salt (mM)**	**Sucrose (%)**	**Hemin (µM)**	**Other**	**Age**	**Atmosphere**	**Shaking (rpm)**	**Biofilm formation**	**eDNA**	**G4**	**Z-DNA**	**B-DNA**	**Replicated**
**1**	*S. mutans* DSM 20523	0.2	TSB	100 (NaCl)	1	10		48 h	M	150	Y		W		W	
**2**	*S. mutans* DSM 20523	0.2	BHI	100 (NaCl)	1	10		48 h	M	150	Y		Y	Y	Y	N
**3**	*S. mutans* DSM 20523	0.2	BHI	100 (NaCl)	1	10	24 h BHI + 24 h sterile saliva	48 h	M	150	Y		N		N	
**4**	*S. mutans* DSM 20523	0.6	BHI	100 (NaCl)	1	10	24 h BHI + 24 h sterile saliva	48 h	M	150	Y		N		Y	
**5**	*S. mutans* DSM 20523	0.2	BHI	100 (NaCl)	1	10	2 h Cm stress (10 µg/ml) after 24 h	48 h	M	150	Y		Y		Y	
**6**	*S. mutans* DSM 20523	0.2	BHI	100 (NaCl)	1	10		24 h	M	150	W		N		Y	
**7**	*S. mutans* DSM 20523	0.2	BHI	100 (NaCl)	0.5	10		24 h	M	150	W		N		Y	
**8**	*S. mutans* DSM 20523	0.2	BHI	100 (NaCl)	0.25	10		24 h	M	150	W		N		Y	
**9**	*S. mutans* DSM 20523	0.2	BHI	100 (NaCl)	0.25	10	Cm (0.25 µg/ml)	24 h	M	150	W		W		Y	
**10**	*S. mutans* DSM 20523	0.2	BHI	0.1 ($CaCl_2$)	0.25	10		48 h	AN	150	Y		W		Y	
**11**	*S. mutans* DSM 20523	0.2	BHI	0.1 ($CaCl_2$)	0.25	10		48 h	M	150	Y		W		Y	
**12**	*S. mutans* DSM 20523	0.2	BHI	0.1 ($CaCl_2$)	0.25	10		48 h	AN	150	Y		W		Y	
**13**	*S. mutans* DSM 20523	0.2	BHI	100 (NaCl)	0.25	10	Acetic acid (25 mM)	48 h	M	150	Y		N		W	
**14**	*S. mutans* DSM 20523	0.2	BHI	100 (NaCl)	0.25	10	Acetic acid (25 mM)	48 h	AN	150	Y		W		Y	

**15**	*S. mutans* DSM 20523	0.2	BHI	100 (NaCl)	0.25	10		7 days	M	150	Y		W		Y	
**16**	*S. mutans* DSM 20523	0.02	BHI	100 (NaCl)	0.25	10		48 h	M	150	Y		N		N	
**17**	*S. mutans* DSM 20523	0.002	BHI	100 (NaCl)	0.25	10		48 h	M	150	Y		N		N	
**18**	*S. mutans* DSM 20523	0.2	BHI	100 (NaCl)	1	5		48 h	M	120	Y		N			
**19**	*S. mutans* DSM 20523	0.2	BHI	100 (NaCl)	1	5	CSP (1 μM)	48 h	M	120	W		N			
**20**	*S. mutans* DSM 20523	0.2	BHI	100 (NaCl)	1	5	CSP (1 μM) after 24 h	48 h	M	120	Y		N			
**21**	*S. mutans* DSM 20523	0.2	BHI	100 (NaCl)	1	5	CSP (1 μM) after 24 h	72 h	M	120	Y		N			
**22**	*S. mutans* DSM 20523	0.2	BHI	100 (NaCl)	5	5		48 h	M	120	Y		N			
**23**	*S. mutans* DSM 20523	0.2	BHI	100 (NaCl)	5	5	CSP (1 μM) after 24 h	48 h	M	120	Y		N			
**24**	*S. mutans* DSM 20523	0.2	BHI	100 (NaCl)	10	5		48 h	M	120	Y		N			
**25**	*S. mutans* DSM 20523	0.2	BHI	100 (NaCl)	10	5	CSP (1 μM) after 24 h	48 h	M	120	Y		N			
**26**	*S. mutans* DSM 20523	0.2	BHI	100 (KCl)	1	5		48 h	M	120	Y		N			Y
**27**	*S. mutans* DSM 20523	0.2	BHI	100 (KCl)	1	5	CSP (1 μM) after 24 h	48 h	M	120	Y		Y			N
**28**	*S. mutans* DSM 20523	0.2	BHI	100 (KCl)	1	5		48 h	M	120	Y		N			
**29**	*S. mutans* DSM 20523	0.2	BHI	100 (KCl)	1	5	CSP (1 μM) after 24 h	48 h	M	120	Y		N			
**30**	*S. mutans* DSM 20523	0.2	BHI	100 (KCl)	1	5	CSP (1 μM)	48 h	M	120	Y		N			
**31**	*S. mutans* DSM 20523	0.2	BHI	100 (KCl)	1	5	CSP (1 μM) after 24 h	72 h	M	120	Y		N			
**32**	*S. mutans* DSM 20523	Single colony	BHI	100 (NaCl)	1	5		24 h	AE	0	Y		Y			
**33**	*S. mutans* DSM 20523	Single colony	BHI	100 (NaCl)	1	5		48 h	AE	0	Y		N			

**34**	*S. mutans* DSM 20523	Single colony	BHI agar					72 h	AE	0	Y		Y	N		
**35**	*S. mutans* SK 70	0.2	BHI	100 (NaCl)	1	10		24 h	M	150	Y		W	W	Y	
**36**	*S. mutans* SK 70	0.2	TSB	100 (NaCl)	1	10		48 h	M	150	W		W		W	
**37**	*S. mutans* SK 70	0.2	BHI	100 (NaCl)	1	5		48 h	M	120	Y		N			
**38**	*S. mutans* SK 70	0.2	BHI	100 (NaCl)	1	5	CSP (1 µM)	48 h	M	120	W		N			
**39**	*S. mutans* SK 70	0.2	BHI	100 (NaCl)	1	5	CSP (1 µM) after 24 h	48 h	M	120	W		N			
**40**	*S. mutans* SK 28	0.2	BHI	100 (NaCl)	1	5		48 h	M	120	Y		N			
**41**	*S. mutans* SK 28	0.2	BHI	100 (NaCl)	1	5	CSP (1 µM)	48 h	M	120	W		N			
**42**	*S. mutans* SK 28	0.2	BHI	100 (NaCl)	1	5	CSP (1 µM) after 24 h	48 h	M	120	Y		N			
**43**	*P. gingivalis* ATCC 33277	0.2	Plaq ue	100 mM NaCl	1	5		48 h	AN	120	Y		Y		Y	N
**44**	*P. gingivalis* ATCC 33277	0.2	Plaq ue	100 (KCl)	1	5		48 h	AN	120	Y		Y		Y	
**45**	*P. gingivalis* ATCC 33277	0.2	TSB	100 (NaCl)	1	5	Yeast extract (1 µg/ml), menadione (1 µg/ml)	48 h	AN	120	W	Y				
**46**	*P. gingivalis* ATCC 33277	0.2	BHI	100 (NaCl)	1	5	Yeast extract (1 µg/ml), menadione (1 µg/ml)	48 h	AN	120	W	Y				
**47**	*P. gingivalis* ATCC 33277	0.2	TSB	100 (NaCl)	1	5	Menadione (1 µg/ml)	48 h	AN	120	W	Y				
**48**	*P. gingivalis* ATCC 33277	0.2	BHI	100 (NaCl)	1	5	Menadione (1 µg/ml)	48 h	AN	120	W	Y				
**49**	*P. gingivalis* ATCC 33277	0.2	BHI	100 (NaCl)	1	5	Yeast extract (1 µg/ml), menadione (1 µg/ml)	7 days	AN	120	N		Ye			

**50**	*P. gingivalis* ATCC 33277	0.2	BHI	100 (NaCl)	1	5	Menadione (1 µg/ml)	7 days	AN	120	N		N			
**51**	*P. gingivalis* ATCC 33277	0.2	TSB	100 (NaCl)	1	5	Yeast extract (1 µg/ml), menadione (1 µg/ml)	7 days	AN	120	W		Y			
**52**	*P. gingivalis* ATCC 33277	0.2	TSB	100 (NaCl)	1	5	Menadione (1 µg/ml)	7 days	AN	120	W		N			
**53**	*P. intermedia* ATCC 25611	0.2	Plaque	100 (NaCl)	1	5		48 h	AN	120	W		N		Y	
**54**	*P. intermedia* ATCC 25611	0.2	Plaque	100 (KCl)	1	5		48 h	AN	120	W		Y		Y	
**55**	*S. sanguinis* ATCC 10556	0.2	BHI	100 (NaCl)	1	10		24 h	AE	150	W	Y				
**56**	*S. sanguinis* ATCC 10556	0.2	TSB	100 (NaCl)	1	10		48 h	M	150	Y					
**57**	*S. sanguinis* ATCC 10556	0.2	BHI	100 (NaCl)	1	5		48 h	M	120	Y		N			
**58**	*S. sanguinis* ATCC 10556	0.2	BHI	100 (NaCl)	1	5	CSP (1 µM) after 24 h	48 h	M	120	Y		N			
**59**	*S. sanguinis* SK 150	0.2	BHI	100 (NaCl)	1	10		24 h	AE	150	W	Y				
**60**	*S. oralis* NCTC 7864	0.2	BHI	100 (NaCl)	1	10		24 h	AE	150	W	Y				
**61**	*S. oralis* NCTC 7864	0.2	TSB	100 (NaCl)	1	10		48 h	M	150	Y					
**62**	*S. oralis* NCTC 7864	0.2	BHI	100 (NaCl)	1	5		48 h	M	120	Y		N			
**63**	*S. oralis* NCTC 7864	0.2	BHI	100 (NaCl)	1	5	CSP (1 µM) after 24 h	48 h	M	120	Y		N			
**64**	*S. oralis* SK 248	0.2	BHI	100 (NaCl)	1	10		24 h	AE	150	W	W				
**65**	*S. gordonii* ATCC 10558	0.2	BHI	100 (NaCl)	1	10		24 h	AE	150	W	Y				
**66**	*S. gordonii* ATCC 10558	0.2	BHI	100 (NaCl)	1	5		48 h	M	120	Y		N			
**67**	*S. gordonii* ATCC 10558	0.2	BHI	100 (NaCl)	1	5	CSP (1 µM) after 24 h	48 h	M	120	Y		N			

**68**	*S. mitis* SK 024	0.2	BHI	100 (NaCl)	1	10		24 h	AE	150	W	Y				
**69**	*S. mitis* SK 024	0.2	TSB	100 (NaCl)	1	10		48 h	M	150	Y					
**70**	*F. nucleatum* ATCC 10953	0.2	Plaque	100 (NaCl)	1	5		48 h	AN	120	N		N		N	
**71**	*F. nucleatum* ATCC 10953	0.2	Plaque	100 (KCl)	1	5		48 h	AN	120	N		N		N	
**72**	*L. paracasei* subsp. *paracasei* DSM 20020	0.2	BHI	100 (NaCl)	1	10		24 h	AE	150	W	W				
**73**	*L. rhamnosus* DSM 20021	0.2	BHI	100 (NaCl)	1	10		24 h	AE	150	N	N				
**74**	Mixed	Plaque	BHI	100 (NaCl)	1	5		72 h	AE	120	Y	Y	W	N		
**75**	Mixed	Plaque	BHI	100 (NaCl)	1	5		72 h	AN	120	Y	Y	N	N		
**76**	Mixed	Plaque	TSB	100 (NaCl)	1	5 uM		72 h	AE	120	Y	Y				
**77**	Mixed	Plaque	TSB	100 (NaCl)	1	5 uM		72 h	AN	120	Y	Y				
**78**	Mixed	Plaque	BHI	100 (NaCl)	1	5 uM		7 days	AE	120	Y		Y	Y		N
**79**	Mixed	Plaque	BHI	100 (NaCl)	1	5 uM		7 days	AN	120	Y		N	N		
